# SNARE SYP132 mediates divergent traffic of plasma membrane H^+^-ATPase AHA1 and antimicrobial PR1 during bacterial pathogenesis

**DOI:** 10.1093/plphys/kiac149

**Published:** 2022-03-28

**Authors:** Guillermo Baena, Lingfeng Xia, Sakharam Waghmare, Rucha Karnik

**Affiliations:** Plant Science Group, Laboratory of Plant Physiology and Biophysics, Institute of Molecular, Cell and Systems Biology, College of Medical, Veterinary and Life Sciences, University of Glasgow, Bower Building, Glasgow G12 8QQ, UK; Plant Science Group, Laboratory of Plant Physiology and Biophysics, Institute of Molecular, Cell and Systems Biology, College of Medical, Veterinary and Life Sciences, University of Glasgow, Bower Building, Glasgow G12 8QQ, UK; Plant Science Group, Laboratory of Plant Physiology and Biophysics, Institute of Molecular, Cell and Systems Biology, College of Medical, Veterinary and Life Sciences, University of Glasgow, Bower Building, Glasgow G12 8QQ, UK; Plant Science Group, Laboratory of Plant Physiology and Biophysics, Institute of Molecular, Cell and Systems Biology, College of Medical, Veterinary and Life Sciences, University of Glasgow, Bower Building, Glasgow G12 8QQ, UK

## Abstract

The vesicle trafficking SYNTAXIN OF PLANTS132 (SYP132) drives hormone-regulated endocytic traffic to suppress the density and function of plasma membrane (PM) H^+^-ATPases. In response to bacterial pathogens, it also promotes secretory traffic of antimicrobial pathogenesis-related (PR) proteins. These seemingly opposite actions of SYP132 raise questions about the mechanistic connections between the two, likely independent, membrane trafficking pathways intersecting plant growth and immunity. To study SYP132 and associated trafficking of PM H^+^-ATPase 1 (AHA1) and PATHOGENESIS-RELATED PROTEIN1 (PR1) during pathogenesis, we used the virulent *Pseudomonas syringae* pv. *tomato* DC3000 (*Pst* DC3000) bacteria for infection of Arabidopsis (*Arabidopsis thaliana*) plants. SYP132 overexpression suppressed bacterial infection in plants through the stomatal route. However, bacterial infection was enhanced when bacteria were infiltrated into leaf tissue to bypass stomatal defenses. Tracking time-dependent changes in native AHA1 and SYP132 abundance, cellular distribution, and function, we discovered that bacterial pathogen infection triggers AHA1 and SYP132 internalization from the plasma membrane. AHA1 bound to SYP132 through its regulatory SNARE Habc domain, and these interactions affected PM H^+^-ATPase traffic. Remarkably, using the Arabidopsis *aha1* mutant, we discovered that AHA1 is essential for moderating SYP132 abundance and associated secretion of PR1 at the plasma membrane for pathogen defense. Thus, we show that during pathogenesis SYP132 coordinates AHA1 with opposing effects on the traffic of AHA1 and PR1.

## Teaser

Coordination between SNARE SYP132 and plasma membrane H^+^-ATPase AHA1 moderates SNARE abundance during pathogenesis with opposing effects on trafficking of AHA1 and antimicrobial pathogenesis-related protein 1.

## Introduction

In plants, plasma membrane (PM) H^+^-ATPases are primary transporters which mediate proton extrusion across the cell membrane, energizing osmotic solute transport, altering apoplast pH, and promoting plastic cell expansion for “acid growth” (Michelet and Boutry, 1995; Sze et al., 1999; Hager, 2003). The activity of PM H^+^-ATPases in guard cells contributes to stomatal movements influencing gas exchange, transpirational water loss, and the entry of microbial pathogens into the plant (Blatt, 2000; Sutter et al., 2007; Keinath et al., 2010; Elmore and Coaker, 2011; Jezek and Blatt, 2017). Thus, PM H^+^-ATPases govern basic aspects of cell expansion, growth, and immunity, underpinning physiological, developmental, and adaptive responses of the plant (Klejchova et al., 2021).

PM H^+^-ATPase activity is regulated by posttranslational modifications (Michelet and Boutry, 1995; Sze et al., 1999). In parallel, membrane traffic modulates the availability of these transporters at the plasma membrane for active function (Hager, 2003; Hashimoto-Sugimoto et al., 2013; Xia et al., 2019). Recent studies demonstrate that SYNTAXIN OF PLANTS132 (SYP132), a soluble *N*-ethylamide-sensitive factor attachment protein receptor (SNARE) protein, promotes PM H^+^-ATPase traffic (Xia et al., 2019, 2020). Thereby, over-expression of the SYP132 reduces PM H^+^-ATPase density and suppresses plant growth (Richardson, 2019; Xia et al., 2019, 2020).

In plants, as in other eukaryotes, SNAREs mediate vesicle trafficking. The fusion of cargo-containing vesicles at the target membrane is SNARE-assisted and is driven by the formation of SNARE core-complexes which include membrane-localized target (t-) and vesicle localized (v-) SNAREs (Sanderfoot et al., 2000; Jahn and Scheller, 2006; Bassham and Blatt, 2008). The SNAREs of plants also play specialized roles in maintaining cellular homeostasis and facilitating plant nutrition, growth, and immunity (Sanderfoot et al., 2000; Pratelli et al., 2004; Karnik et al., 2017). Functional regulation of the syntaxin SNAREs is stringent and is facilitated by a well-conserved domain-oriented structure including an unstructured N-terminus, a helical Habc domain, the conserved Qa-SNARE domain, and a C-terminal transmembrane domain which is essential for membrane localization (Jahn and Scheller, 2006; Han et al., 2017). The Habc helices of the syntaxins can fold back, masking the Qa-SNARE motif, for control of interactions with cognate SNARE and non-SNARE partners (Sanderfoot et al., 2000; Jahn and Scheller, 2006; Bassham and Blatt, 2008; Sudhof and Rothman, 2009; Karnik et al., 2013, 2015, 2017; Zhang et al., 2019).

In Arabidopsis (*Arabidopsis thaliana*), the t-SNAREs (or syntaxins), SYP121 (Syntaxin of Plants121 or SYNTAXIN RELATED PROTEIN1/PENETRATION1 [SYR1/PEN1]), SYP122, (SYNTAXIN OF PLANTS122) and SYP132 occur throughout the plant, and together they facilitate a bulk of cellular vesicle traffic at the plasma membrane (Enami et al., 2009; El Kasmi et al., 2013; Karnik et al., 2013; Ichikawa et al., 2014; Karnik et al., 2015). These three homologous SNAREs share over 60% sequence similarity and have overlapping as well as distinct functions (Waghmare et al., 2018). For example, both SYP121 and SYP122 are involved in defense against fungal pathogens. SYP121 facilitates focal secretory traffic for penetration resistance against powdery mildew fungus (Assaad et al., 2004; Kwon et al., 2008; Nielsen and Thordal-Christensen, 2012). Additionally, SYP121 is important in abiotic stress tolerance (Eisenach et al., 2012) but it has no apparent role in bacterial pathogenesis (Zhang et al., 2007; Kwon et al., 2020). SYP122 facilitates secretory traffic at the plasma membrane in response to bacterial elicitors (Nuhse et al., 2003; Elmore et al., 2012), contributes to salicylic acid and jasmonic acid signaling (Assaad et al., 2004; Zhang et al., 2008), and is primarily regulated by posttranslational modifications (Nuhse et al., 2003).

Together with the Syntaxin SYP111 (SYNTAXIN OF PLANTS111 or KNOLLE), SYP132 drives secretory traffic at the phragmoplast for cytokinesis, which makes it essential for plant survival. Indeed, *syp132* null mutants are not viable (Sanderfoot et al., 2000; Enami et al., 2009; Park et al., 2018). In the vegetative plant SYP132 is localized to the plasma membrane (Ichikawa et al., 2014; Xia et al., 2019). The growth hormone auxin downregulates *SYP132* expression and suppresses AHA1 internalization from the plasma membrane (Xia et al., 2019). Conversely, for defense against bacterial pathogens, SYP132 is recruited for secretion of antimicrobial PATHOGENESIS-RELATED PROTEIN1 (PR1) to the apoplast (Kalde et al., 2007; Hussan et al., 2020). In a nutshell, all existing knowledge on one hand attributes SYP132-assisted internalization as an essential component for PM H^+^-ATPase regulation, but on the other hand, it implicates SYP132-mediated secretory traffic as a key player for immune responses against bacterial pathogens. Thus, while SYP132 is clearly at the center of growth and immune responses in plants, its seemingly opposing actions on membrane traffic pose a challenge to the understanding of its function in the plant.

Here, we investigated the PM H^+^-ATPase-SYP132 trafficking nexus and its impact on antimicrobial PR1 secretion using the Arabidopsis*–Pseudomonas syringae* pv. tomato DC3000 (*Pst* DC3000) pathosystem as a model (Xin and He, 2013). Time-dependent changes in bacterial proliferation were measured and native PM H^+^-ATPase 1 (AHA1) and SYP132 protein densities at the plasma membrane were tracked using biochemical and physiological analysis. We found that during pathogenesis coordinated traffic of AHA1 and SYP132 from the plasma membrane is governed by their binding. SYP132 and AHA1 trafficking affects functions of these proteins at the plasma membrane, facilitating stomatal defense and resistance to bacterial pathogen entry into the plant, but moderating postinfection immune responses. Thus, we uncover a mechanism for SNARE-regulation by AHA1 involving membrane traffic of SYP132 itself, governed unusually by protein abundance that governs defense-related secretory traffic in plants.

## Results

Secretory traffic at the plasma membrane associated with the SNARE SYP132 is deemed essential for postpenetration plant immunity (Kalde et al., 2007; Hussan et al., 2020). Silencing of SYP132 expression inhibits the accumulation of antimicrobials, including the PR1 proteins in the apoplast and compromises disease resistance (Kalde et al., 2007), yet the mechanics of SYP132-regulation during pathogenesis is undefined.

### SYP132 contributes to suppression of bacterial infection via stomatal entry

**Figure 1:**
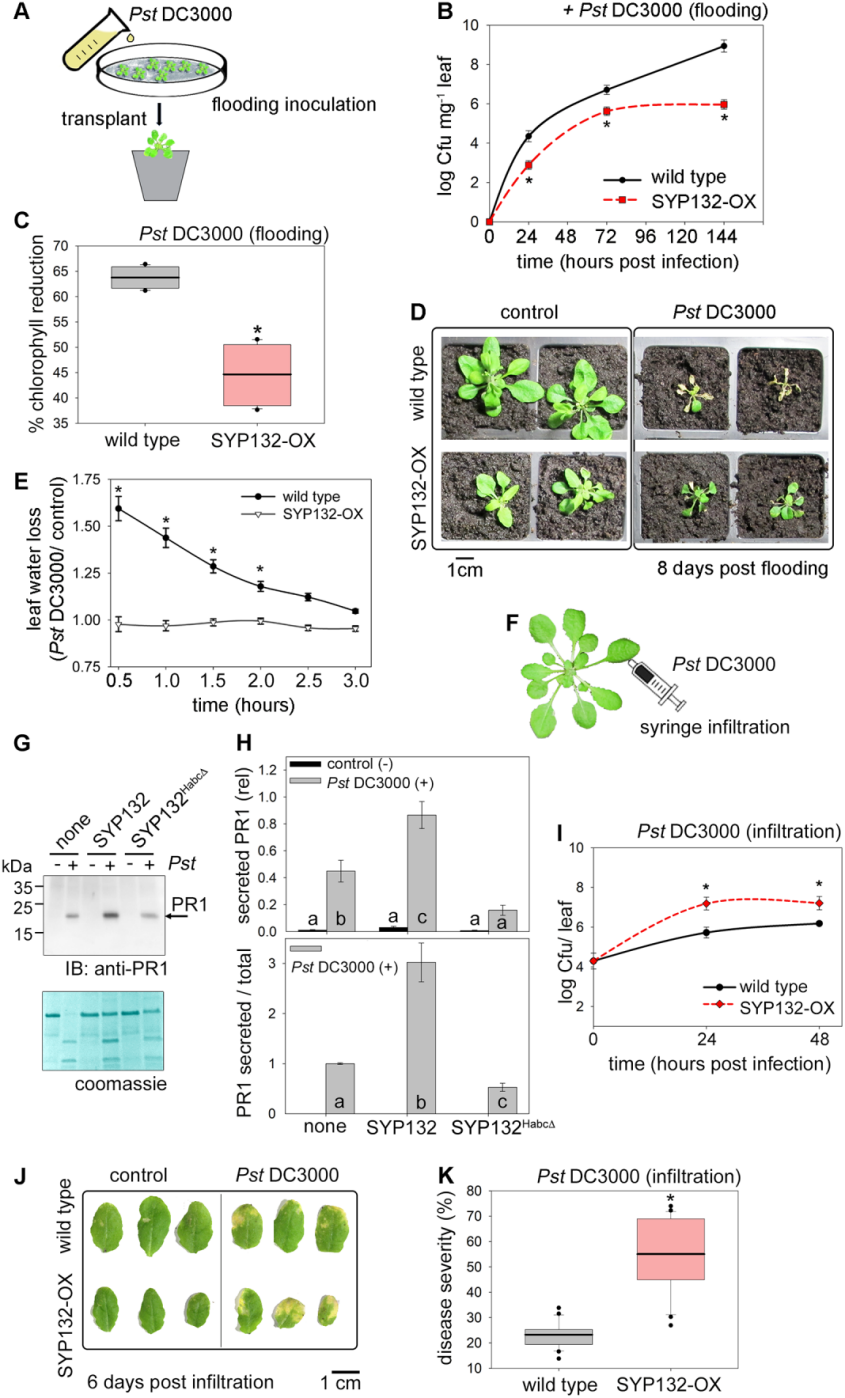
SYP132 promotes stomatal defenses but compromises postinfection immunity (also see Supplemental Figure S1). A, Schematic for flood-inoculation of plants with bacterial pathogens for infection via stomatal route. B, Mean ± se Cfu mg^−1^ in wild-type and p*CaMV* 35S: RFP-SYP132 Arabidopsis over-expressing SYP132 (SYP132-OX) flood-inoculated with *Pst* DC3000 inoculum at 5 × 10^7^ Cfu mL^−1^ measured at 24-h **Figure 1** (continued) intervals postinfection for 144 h and plotted on a logarithmic scale. Statistically significant differences assessed using Mann–Whitney–Wilcoxon test are indicated with “*” for each timepoint (*P* < 0.01), N ≥ 3 independent experiments with ≥9 plants. C, Wild-type and SYP132-OX Arabidopsis were flood-inoculated with pathogens *Pst* DC3000 at 5 × 10^7^ Cfu mL^−1^ or 10 mM MgCl_2_, 0.025% v/v Silwet-L77 buffer (control). Box plots with error bars depict percent reduction in chlorophyll pigments for pathogen challenged leaves relative to buffer. Thin horizontal lines represent the median, bold horizontal lines represent the mean, box limits show the 25th and 75th percentiles. Outliers that exceed their whisker range (1.5× interquartile range) are represented by dots. Statistically significant differences assessed using Mann–Whitney–Wilcoxon test are indicated with “*” (*P* < 0.001), *N* = 3, using ≥6 plants per each experiment. Note: horizontal lines for mean and median are overlapped. D, Representative photographs of wild-type and SYP132-OX Arabidopsis flood-inoculated with buffer (control) or *Pst* DC3000 inoculum at 5 × 10^7^ Cfu mL^−1^ and grown on soil for 8 d are shown. Images are aligned digitally for comparisons. *N* = 3. Scale bar = 1 cm (accurate for each image). E, Mean ± se values for leaf water loss over time (hours), in leaf dehydration assays on excised leaves from wild-type and SYP132-OX Arabidopsis. Leaf water loss was calculated as change in leaf weight due to dehydration following treatment with *Pst* DC3000 relative to buffer (control). Statistically significant differences assessed using Mann–Whitney–Wilcoxon test are indicated with “*” (*P* < 0.001), *N* ≥ 3. Data are from ≥9 plants and ≥6 measurements in each experiment. F, Schematic for bacterial infection of leaves by syringe infiltration bypassing stomata. G, Representative immunoblot shows PR1 band at ∼20 kDa detected using anti-PR1 antibodies (top panel) of apoplast flush derived from *N. benthamiana* leaves treated with buffer (control) or with *Pst* DC3000 in buffer for 48 h. Leaves were untransformed (none), or transiently transformed to express RFP-fused to the full length SYP132 (SYP132) or to the dominant negative, so-called Sp3-fragment (SYP132^HabcΔ^). The Coomassie-stained membrane (bottom panel) shows total protein per lane. Note, the nature of the upper band in the Coomassie is unknown, but the pattern appeared in every experiment). Black lines (left) indicate position of molecular mass markers, and black arrows (right) indicate expected band positions. PR1 and SNARE expression was verified by corresponding immunoblot analysis of total leaf tissue (see Supplemental Figure S1H). H, Graphs for mean ± se for secreted PR1 in apoplast flush normalized to total protein in each lane (upper panel), and for secreted PR1 relative to total PR1 in the pathogen treated samples (lower panel). Black bars represent data from control (buffer, − *Pst* DC3000), and grey bars represent data from *Pst* DC3000 (+) infected leaves. Letters indicate statistically significant differences assessed using ANOVA (*P* < 0.001), *N* = 3 with ≥6 plants in each experiment. I, *Pst* DC3000 population in wild-type or SYP132-OX Arabidopsis following infiltration with 10 μL *Pst* DC3000 inoculum at 2.5 × 10^5^ Cfu mL^−1^. Graphs are mean ± se*Pst* DC3000 Cfu per leaf plotted on a logarithmic scale over hour (s) postinfection. Statistically significant differences assessed using Mann–Whitney–Wilcoxon test are indicated with “*” after comparing Cfu counts for different Arabidopsis lines at each timepoint (*P* < 0.001), *N* = 3 with ≥6 plants in each experiment. J, Representative photographs of wild-type and SYP132-OX Arabidopsis infiltrated with 10 μL *Pst* DC3000 inoculum at 2.5 × 10^5^ Cfu mL^−1^ or buffer (control) at 0 and 96 h postinfection are shown. Images are aligned digitally. *N* = 3. Scale bar = 1 cm (accurate for each image). K, Box plot with error bars depicting % disease severity in wild-type and SYP132-OX Arabidopsis after 72 h following infiltration with 10 μL *Pst* DC3000 inoculum at 2.5 × 10^5^  Cfu mL^−1^. Thin horizontal lines represent the median, bold horizontal lines represent the mean, box limits show the 25th and 75th percentiles. Outliers that exceed their whisker range (1.5× interquartile range) are represented by dots. “*” indicates statistical significance using Mann–Whitney–Wilcoxon test (*P* < 0.01), *N* = 3 with ≥6 plants in each experiment. Note: horizontal lines for mean and median are overlapped.

To assay bacterial proliferation in leaf tissue following infection via the stomatal route, we followed standard flood-inoculation of Arabidopsis seedlings (Figure 1A) using *Pseudomonas syringe* (*Pst* DC3000) bacteria suspended in buffer (10 mM MgCl_2_, 0.025% Silwet-L77, control) for infection, and the counting of bacterial colony-forming units (Cfu) to determine bacterial proliferation (Tornero and Dangl, 2001; Ishiga et al., 2011; Xin and He, 2013). Bacterial infection was inoculum dose-dependent over the first 48 h postinfection. Thereafter bacterial growth saturated (Supplemental Figure S1A) and the plants appeared diseased (Supplemental Figure S1B).

The *syp132* null mutant plants are not viable, and near-complete silencing of *SYP132* expression causes growth defects and blocks secretory traffic associated with this SNARE (Sanderfoot et al., 2000; Kalde et al., 2007; Enami et al., 2009; Park et al., 2018). A near-complete suppression of SYP132 expression, such as using SYP132 RNAi knockdown approach, would hence be unlikely to reveal more than the previous silencing study for SYP132 (Kalde et al., 2007); and a complete suppression would be useless as a means of examining SYP132 regulation. The haplo-impaired *syp132*^T^ line has reduced *SYP132* transcripts compared with wild-type plants (Park et al., 2018; Xia et al., 2019). *syp132*^T^ plants show an increase in PM H^+^-ATPase abundance and activity but these marginal increases do not translate into appreciable changes in plant growth (Xia et al., 2019). We suspected therefore that partial suppression of *SYP132*, such as exhibited by the haplo-impaired *syp132*^T^ line, would likely be difficult to interpret. In p*CaMV* 35S: RFP-SYP132 Arabidopsis over-expressing RED FLUORESCENT PROTEIN (RFP)-tagged SYP132 (SYP132-OX; Xia et al., 2019, 2020) the background *SYP132* expression is almost 12-folds higher compared with the wild-type plants, thus opposing the natural order (Xia et al., 2019, 2020). Therefore, to study this SNARE during pathogenesis, we used SYP132-OX plants as a tool, and for comparing with wild-type and *aha1-7* mutants.

Previously, we showed that SYP132 downregulates the PM H^+^-ATPase activity and that SYP132 over-expression inhibits shoot growth and reduces steady-state stomatal aperture (Xia et al., 2019). Hence, it was anticipated that overexpressing the SNARE would suppress bacterial entry into the plant via the stomatal route and reduce infection of bacterial pathogens inoculated onto the leaf surface. As expected, Arabidopsis overexpressing SYP132 (SYP132-OX) developed fewer bacterial infections compared with wild-type plants (Figure 1B). Pathogen infection (Supplemental Figure S1C) was similar in both of the independently transformed SYP132-OX lines previously described (Xia et al., 2019), so in subsequent experiments we pooled the data from these two lines. As a measure of plant response to disease (Harris and Baulcombe, 2015), we examined foliar chlorophyll content. Chlorophyll content in wild-type and SYP132-OX plants was similar (Supplemental Figure S1D). Bacterial pathogen infection severely reduced chlorophyll content in the wild-type plants. However, in SYP132-OX plants change in chlorophyll content was significantly lower suggesting that the effect of pathogens was much less pronounced (Figure 1C). Pathogen challenge severely restricted growth and survival of wild-type plants. In comparison, SYP132-OX Arabidopsis appeared to have survived pathogen infection, although growth was compromised (Figure 1D).

Several pathogens, including the *Pst* DC3000, counter stomatal defenses by manipulating stomatal aperture by modulating PM H^+^-ATPase activity to re-open the stoma (Melotto et al., 2006; Frei dit Frey and Robatzek, 2009; Dodds and Rathjen, 2010; Elmore and Coaker, 2011; Melotto et al., 2017). To probe the influence of bacteria on stomatal behavior in SYP132-OX Arabidopsis, we measured the dehydration of excised leaves over 3 h following flood-inoculation with *Pst* DC3000 and compared these data with leaves treated with buffer (control). Following bacterial challenge, the stomatal re-opening should lead to rapid leaf water loss. Indeed, wild-type Arabidopsis leaves exhibited significant water loss within 1 h after bacterial challenge; by comparison, water loss from leaves of SYP132-OX plants was very low (Figure 1E). Together with the infection results, these observations suggest that SYP132 contributes to stomatal closure mechanisms promoting plant stomatal defenses.

### SYP132 negatively affects postentry bacterial infection, but mediates antimicrobial secretion

To examine bacterial infection postentry, we used standard syringe infiltration assays (Figure 1F) that fill the internal air space of the leaf with bacterial inoculum, thus by-passing stomatal defenses. Leaves from 4- to 5-week-old, soil-grown Arabidopsis (Yao et al., 2013; Rufian et al., 2019) were injected with buffer (10 mM MgCl_2_; control) or with *Pst* DC3000 inoculum in buffer, and bacterial counts were determined (Shen et al., 2013; Melotto et al., 2017) at intervals over 72 h. Bacterial multiplication in infiltrated leaves depended on the initial *Pst* DC3000 inoculum concentration in the early phases of infection (0–48 h) after which bacterial growth saturated (Supplemental Figure S1E) and the infected leaves developed chlorosis (Supplemental Figure S1F). Disease severity was calculated as percentage of leaf area with chlorotic changes using the ImageJ software (see “Materials and methods”). We observed elevation in disease severity, corresponding to increasing *Pst* DC3000 inoculum concentrations and bacterial infections (Supplemental Figure S1G). These data indicate that active microbial growth can be tracked for the first 48 h in this pathosystem following bacterial infection of Arabidopsis by infiltration.

Defense responses to bacterial infection include secretion of antimicrobial PR1 peptides to the apoplast, for which the SNARE SYP132 is essential (Kalde et al., 2007). To test if block of SYP132 function interferes with PR1 secretory traffic, we used SYP132^HabcΔ^, the so-called Sp3-fragment. SYP132^HabcΔ^ lacks the C-terminal membrane anchor and the conserved H3 domain essential for SNARE core complex assembly. SYP132^HabcΔ^ can bind with the full-length SYP132 and perturb secretory traffic associated with this SNARE, but without influencing parallel secretory trafficking pathways involving the SNARE SYP121/SYP122 nexus (Kargul et al., 2001; Xia et al., 2019). *Nicotiana benthamiana* leaves were transformed to transiently overexpress SYP132^HabcΔ^ and infiltrated with buffer as a control or with *Pst* DC3000 inoculum to evoke PR1 expression. The apoplast was flushed and collect 48 h postinfection, and secreted PR1 was detected using immunoblot with anti-PR1 antibodies. In parallel, total PR1 and RFP-tagged SYP132 and SYP132^HabcΔ^ density in the leaf tissue was verified by immunoblot (Supplemental Figure S1H). We observed that infection of *Pst* DC3000 evoked PR1 accumulation in the apoplast. However, PR1 secretion in pathogen-challenged plants was significantly reduced upon SYP132^HabcΔ^ expression compared to the untransformed leaves, leaves over-expressing the full-length SYP132, and relative to total PR1 in infected tissue (Figure 1, G and H). These data confirmed that the block of SYP132 function inhibits PR1 secretion into the apoplast.

Given the observations above, we expected that SYP132-OX plants would have enhanced defense related secretory trafficking and therefore should resist *Pst* DC3000 proliferation. Instead, we found that bacterial counts were significantly higher in SYP132-OX leaves compared with the wild-type Arabidopsis (Figure 1I). The proliferation of bacteria in both of the independently transformed SYP132-OX lines was similar following infiltration of the pathogens (Supplemental Figure S1I). As the two lines yielded equivalent results, we pooled the data from the two lines in subsequent experiments. SYP132-OX plants showed enhanced chlorosis (Figure 1J) and increased disease severity (Figure 1K), suggesting increased sensitivity to bacterial infection compared with the wild-type plants. Thus, once the stomatal barriers were bypassed, SYP132 overexpression appeared to promote postentry bacterial infection. These observations are unlikely to be just a secondary effect of over-expression as they contradict the enhanced stomatal defense phenotype associated with this SNARE, attributed to endocytosis of the PM H^+^-ATPases (Xia et al., 2019). We asked therefore, how is PM H^+^-ATPase trafficking regulated following bacterial infection and how might it be connected to SYP132-dependent secretion.

### SYP132 and AHA1 redistribute from the PM during bacterial pathogenesis

The SNARE SYP132 promotes AHA1 endocytosis from the plasma membrane and the hormone auxin regulates SYP132 expression to control AHA1 traffic (Xia et al., 2019). SYP132 itself is predominantly localized to the plasma membrane, but it is also known to accompany PM H^+^-ATPase AHA1 to internal membranes (IMs; Enami et al., 2009; Hashimoto-Sugimoto et al., 2013; Xia et al., 2019). Therefore, we asked if bacterial infection might influence co-ordinate traffic, altering cellular redistributions of the AHA1 and SYP132 proteins.

**Figure 2:**
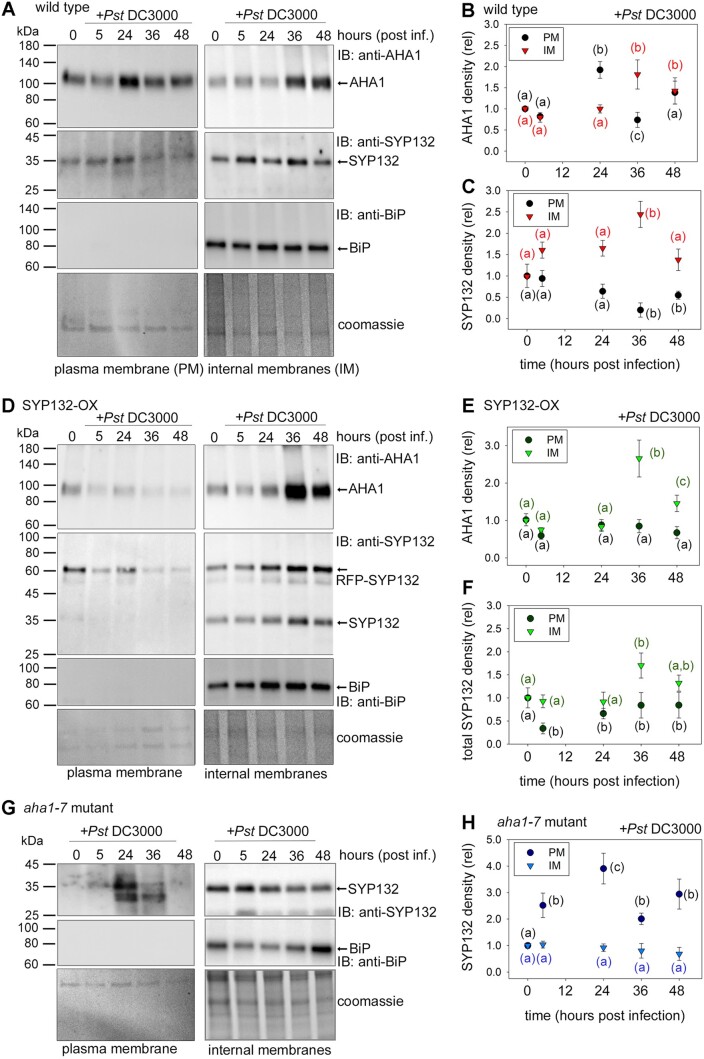
Time-sensitive changes in AHA1 and SYP132 localization occur during bacterial pathogenesis (also see Supplemental Figures S2 and S3). A, D, and G, Immunoblot analysis of Arabidopsis PM and IM fractions resolved using SDS-PAGE following infiltration with 10 μL *Pst* DC3000 inoculum at 2.5 × 10^5^ Cfu mL^−1^ in buffer (10 mM MgCl_2_), at different hours postinfection. Native AHA1 at ∼100 kDa (first panels) was detected using anti-AHA1 antibodies, and total SYP132 was detected at ∼35 kDa for native SYP132, and at ∼61 kDa RFP-SYP132 (second panels) using anti-SYP132 antibodies. Lumen-BiP at ∼74 kDa (third panels), a marker for IMs was detected using anti-BiP antibodies. Representative immunoblots of **Figure 2** (continued) PM and IM fractions from wild-type Arabidopsis (A), p*CaMV* 35S: RFP-SYP132 Arabidopsis over-expressing SYP132 (SYP132-OX) (B) and Arabidopsis *aha1-7* mutant (C) are shown. Purity of the PM fractions was estimated at >99% using BiP bands as reference. Total protein was detected using Coomassie stained immunoblot membrane (fourth panels) and used for quantitative analysis. Black lines (left) indicate positions of molecular mass markers, and black arrows (right) indicate expected band positions. B, C, E, F, and H, Mean ± se protein levels for each time point (hours postinfection) in different Arabidopsis lines. Plots include protein densities in PM (circles) and IMs (triangles) in Arabidopsis; wild-type plants AHA1 (B), SYP132 (C), SYP132-OX lines AHA1 (E), total SYP132, adding native and RFP-SYP132 (F), and *aha1-7* mutant plants SYP132 (H). Values were obtained from densitometric analysis of immunoblots, normalized total protein detected using Coomassie stain, relative to buffer treated control (time = 0). Letters denote statistically significant differences determined using ANOVA (*P* < 0.05), *N* ≥ 3, with ≥6 plants for each timepoint, per experiment. Note: About 10-fold more total protein was loaded for IM fractions than for PM fractions, precluding a direct comparison between PM and IM samples.

Changes in AHA1 and SYP132 protein densities during pathogenesis were determined for PM and IM fractions in Arabidopsis wild-type, SYP132-OX, and *aha1-7* mutant lines. Leaf tissue collected at different time intervals from *Pst* DC3000 infected plants was used to purify PM and IM fractions with an aqueous two-polymer phase system (Sutter et al., 2007; Xia et al., 2019) and subjected to immunoblot analysis. Custom synthesized anti-AHA1 and anti-SYP132 antibodies were used to track native AHA1 and SYP132 protein levels in each plant line relative to time zero (Figure 2), each verified for specificity in native protein detection (Supplemental Figure S2). As a control for these experiments, immunoblot analysis was carried out to detect the lumen-binding protein (BiP), an endoplasmic reticulum resident protein, which marks IMs (Figure 2, A, D, andG). BiP was detected in IM fractions but was virtually absent in PM fractions indicating high purity (>99%).

In the wild-type plants, the density of AHA1 proteins in PM fractions increased transiently following bacterial infection and then declined before returning to normal levels. The reduction of AHA1 population at the PM was complemented with increased levels of the protein within IMs suggesting that a bulk of AHA1 may be relocalized to IMs at 36 h postinfection (Figure 2, A and B). In parallel, SYP132 levels in PM fractions reduced significantly following pathogen infection but increased in the IMs of the wild-type plants (Figure 2, A and C). These data suggest that together with AHA1, abundance of SYP132 at the PM is modulated during pathogenesis, with significant change occurring around 36 h postinfection.

In a similar set of experiments, but using SYP132-OX plants, we tested if increased SNARE abundance impacted re-distribution of AHA1 and SYP132 following *Pst* DC3000 challenge. AHA1 density at the PM remained stable following *Pst* DC3000 infection in the SYP132-OX plants, but AHA1 protein levels in the IMs increased by almost three-fold at 36 h postinfection (Figure 2, D and E). Previously, we had found that PM H^+^-ATPase endocytosis is enhanced in these transgenic lines (Xia et al., 2019). Increased AHA1 abundance in IMs could therefore be attributed, at least in part, for enhanced AHA1 internalization.

In SYP132-OX plants, the total SNARE population includes both the native and RFP-tagged SNARE, so the background SNARE levels are elevated. We found that at 5 h following infection total SYP132 in PM fractions reduced but increased in the IM fractions in the SYP132-OX plants (Figure 2, D and F). Thus, bacterial infection appears to evoke redistribution of both AHA1 and SYP132 from the PM, although biosynthesis could also contribute to the protein densities in the IMs.

To verify if AHA1 and SYP132 traffic from the plasma membrane is influenced by pathogen infection, we used stomatal guard cells as a model. AHA1 is the predominant PM H^+^-ATPase isoform in this cell type (Yamauchi et al., 2016). Confocal images of stomata in Arabidopsis transgenic lines expressing GREEN FLUORESCENT PROTEIN (GFP)-tagged AHA1 and/or RFP-SYP132 driven by the 35S promoter (Xia et al., 2019) were acquired, thus discounting background noise from pathogen-induced changes in native AHA1 and SYP132 expression. Protein expression was verified by immunoblot. At 48 h prior to imaging, leaves were infiltrated with *Pst* DC3000 or with buffer as a control (Supplemental Figure S3, A–I). Protein distributions at cell periphery and interior were quantified using the ImageJ software (see “Materials and methods”). In control conditions, majority of RFP-SYP132 and GFP-AHA1 fluorescence was detected at cell periphery, and only about 20% of the protein population appeared in IMs. These results are consistent with published findings (Reichardt et al., 2011; Ichikawa et al., 2014; Xia et al., 2019). Following *Pst* DC3000 infection the internal/peripheral fluorescence ratio for both RFP-SYP132 and GFP-AHA1 increased by over three-fold compared with the buffer control, attributed to redistribution from the plasma membrane to the cell interior. When the RFP-SYP132 and GFP-AHA1 were co-expressed, cellular redistribution of these proteins was further enhanced by about two-fold following pathogen infection (Supplemental Figure S3, E and F). These data demonstrate that bacterial infection promotes SYP132 redistribution from the cell periphery together with AHA1 and that the abundance of both proteins might affect this traffic. These observations are interesting because they support the possibility that when SYP132 abundance crosses a threshold, dynamic redistribution from the plasma membrane moderates density of this SNARE at the plasma membrane.

We tested if SYP132 trafficking was associated with AHA1 by tracking the redistribution of SYP132 in *aha1-7* mutant Arabidopsis infected with *Pst* DC3000. In this case, we found that native SYP132 levels at the plasma membrane increased over time following pathogen infection, while the density of the SNARE in the IM fractions showed no change in these mutant plants (Figure 2, G and H). Thus, in the presence of AHA1, levels of SYP132 at the plasma membrane are regulated by endocytic traffic of the SNARE itself. Taken together, these data show that during pathogenesis re-distribution of AHA1 and SYP132 to IMs regulates the densities of both these proteins at the plasma membrane and that the trafficking pathways for SYP132 and AHA1 could be coordinated.

### Bacterial infection evokes changes in SYP132 and AHA1 expression

We asked if bacterial pathogen infection leads to changes in the expression of the *SYP132* gene in Arabidopsis. SYP132 is constitutively expressed throughout the plant but is normally low abundant (Enami et al., 2009; Xia et al., 2019). Auxin downregulates *SYP132* expression, promoting the population of H^+^-ATPases at the plasma membrane and enhancing plant growth (Enami et al., 2009; Ichikawa et al., 2014; Xia et al., 2019, 2020). However, when bacteria infect the plant, the demand on SYP132 increases for defense-related functions (Kalde et al., 2007) and the SNARE is required for secretion of antimicrobial PR1 to the apoplast (Figure 1, G and H). Both the major PM H^+^-ATPase isoforms in Arabidopsis, AHA1 and AHA2 (Haruta and Sussman, 2012) are also implicated in plant immune responses (Zhou et al., 2015; Kaundal et al., 2017). Therefore, we tested if expression of all these genes might be regulated following bacterial challenge.

**Figure 3:**
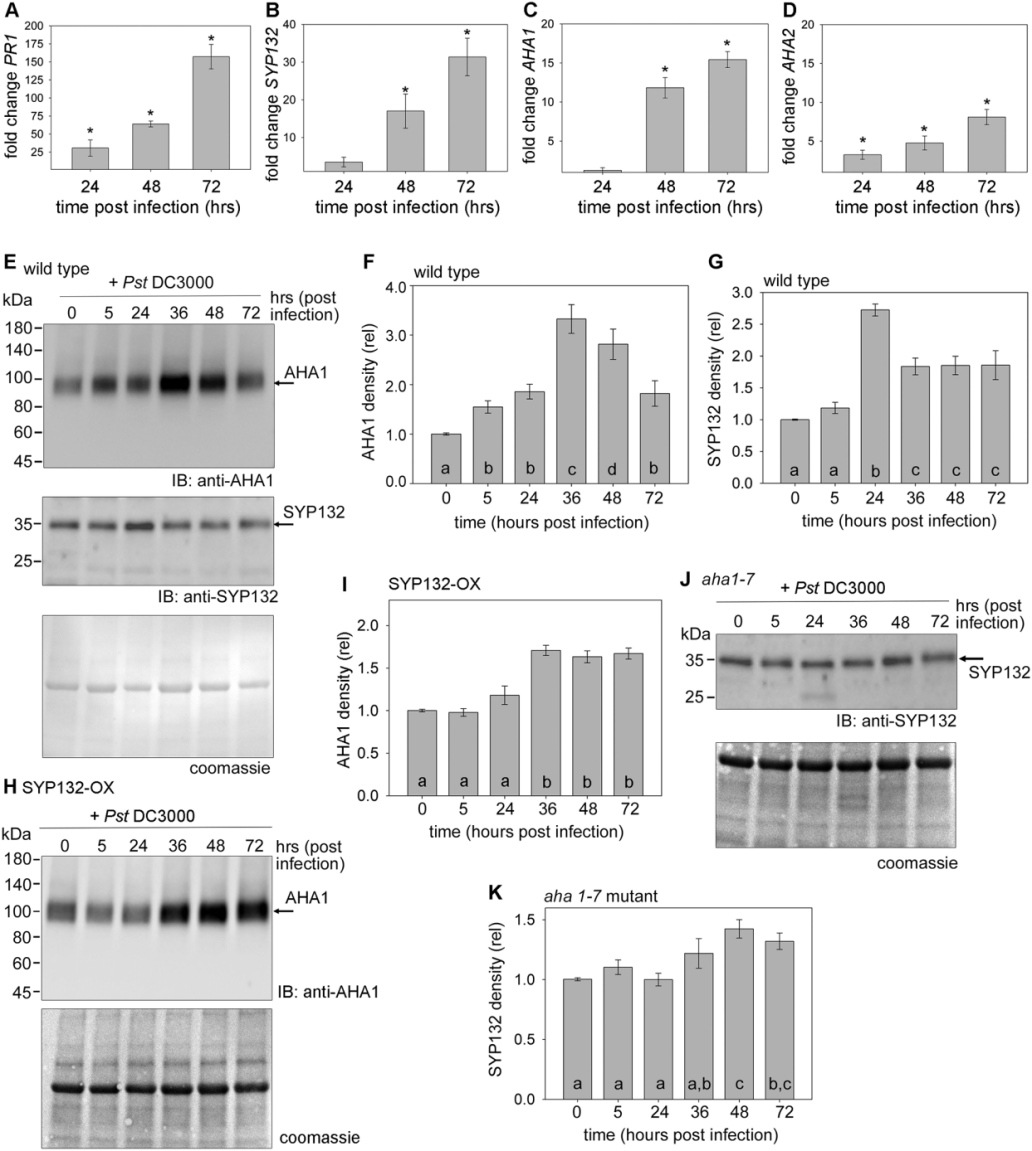
Bacterial infection influences *AHA1* and *SYP132* expression and protein abundance over time (see also Supplemental Figures S2 and S4). A–D, RT-qPCR analysis of gene expression in wild-type Arabidopsis leaves syringe infiltrated with 10 μL *Pseudomonas syringae* (*Pst* DC3000) inoculum at 2.5 × 10^5^ Cfu mL^−1^ in buffer (10 mM MgCl_2_). Fold change in gene *PR1* (A), *SYP132* (B), *AHA1* (C), and *AHA2* (D) transcripts measured relative to mitochondrial 18s rRNA (AtMg01390) calculated (2^−ΔΔCt^ method, Livak and Schmittgen, 2001) relative to buffer at 24-, 48-, and 72-h postinfection. Since the data were either not normally distributed or had unequal variances, a Kruskal–Wallis ANOVA on ranks was undertaken and a pairwise comparison using Dunn’s method was applied. Data are mean ± se values (*P* < 0.001) representative of three biological replicates. Leaves were sampled from ≥3 plants for each timepoint in every experiment. E, H, and J, Representative immunoblots of microsomal membrane proteins resolved on SDS-PAGE to detect native proteins AHA1 at ∼100 kDa or SYP132 at ∼35 kDa using anti-AHA1 and anti-SYP132 antibodies, respectively. Microsomes were extracted from Arabidopsis leaves of wild-type (E), p*CaMV* 35S: RFP-SYP132 Arabidopsis over-expressing SYP132 (SYP132-OX) (H) and *aha1-7* null mutant (J) plants infiltrated with *Pst* DC3000 or with buffer as control (time 0). Total protein in each lane detected using Coomassie stain (bottom panel). Black lines (left) indicate position of molecular mass markers, and black arrows (right) indicate expected band positions. *N* = 3, using ≥5 plants for each timepoint. (Corresponding immunoblots in Supplemental Figure S4, C, E, and G.) F, G, I, and K, Protein levels in wild-type Arabidopsis calculated for AHA1 (F) and SYP132 (G) in wild-type Arabidopsis, for AHA1 in SYP132-OX plants (I) and SYP132 in *aha1-7* mutants (K). Band intensities were measured using ImageJ software, normalized to total protein per lane detected using Coomassie stain, and plotted relative to 10 mM MgCl_2_ treated control (time 0) for each protein. Data are means ± se protein densities. Letters denote statistically significant differences determined using ANOVA (*P* < 0.001), *N* = 3, with ≥5 plants for each timepoint.

Reverse transcription-quantitative PCR (RT-qPCR) analysis was conducted to measure change in transcript levels of *AHA1*, *AHA2*, *SYP132*, and *PR1* in Arabidopsis leaves infiltrated with *Pst* DC3000 compared with mock-infiltrated control (Figure 3, A–D). Using gene-specific primers for the analysis, we observed that *PR1* expression increased by 25-fold 24 h postinfection and was almost 125-fold higher compared with control after 72 h (Figure 3A). In parallel, *SYP132* transcript levels increased sharply as expected and were almost 30-fold higher at 72 h postinfection (Figure 3B). Thus, changes in *SYP132* levels in response to bacteria coincided with increased *PR1* expression.

Initially at 24 h post *Pst* DC3000 infection, no significant changes in *AHA1* transcript levels were observed, but subsequently *AHA1* transcript levels increased sharply and by 72 h postinfection *AHA1* expression was 16-fold higher relative to control (Figure 3C). A three- to six-fold elevation in *AHA2* transcripts was also observed over 72 h following bacterial infection (Figure 3D). Contrary to expectation, the expression of the major PM H^+^-ATPase isoforms *AHA1* and *AHA2* also increased, but the effects of bacteria on *AHA1* transcription were most pronounced.

Native SYP132 and AHA1 protein levels were also tracked in *Pst* DC3000 infected plants by immunoblot analysis of total microsomal fractions. AHA1 and SYP132 levels in untreated wild-type Arabidopsis showed no significant changes over time when compared against mock-infiltrated plants (Supplemental Figure S4, A and B), thus discounting any possible effects of wounding on AHA1 and SYP132 proteins in the leaf tissue. In the wild-type Arabidopsis infiltrated with *Pst* DC3000 (in buffer), both AHA1 and SYP132 expression showed finite changes, coinciding in time, following bacterial infection (Figure 3, E–G). AHA1 density in the wild-type plants was over three-fold higher by 36 h postinfection, and then subsequently reduced but was still two-fold higher at 72 h postinfection relative to time zero (Figure 3, E and F). The total SYP132 protein levels increased sharply by over 2.5-fold at 24 h following bacterial infection and then reduced but remained higher compared to those in mock infiltrated tissue at 0 h (Figure 3G). Thus, our data suggest that both AHA1 and SYP132 protein levels follow a qualitatively similar pattern for change during pathogenesis.

To determine if the changes in AHA1 and SYP132 abundance are co-dependent, we analyzed AHA1 density in SYP132-OX lines (Figure 3, H and I), and conversely SYP132 levels were measured in *aha1-7* null mutant Arabidopsis (Haruta and Sussman, 2012), each following infiltration with *Pst* DC3000 (Figure 3, J and K). In the SYP132-OX plants, a consistent 1.5-fold elevation in AHA1 protein density was observed after 24 h postinfection (Figure 3, H and I). Overexpressing the SNARE elevated background SYP132 abundance by up to three-fold, when compared with native SYP132 levels. The total SYP132 protein levels (native + RFP-SYP132) in these plants remained steady up to 48 h following pathogen infection (Supplemental Figure S4, C and D). Interestingly, however, in *aha1-7* null mutants SYP132 protein levels increased over time in response to *Pst* DC3000 (Figure 3, J and K), instead of decreasing as in wild-type plants (Figure 3, E and G). No obvious change in total AHA protein abundance was observed in the *aha1-7* null mutant in immunoblot analysis using anti-H^+^-ATPase antibodies which bind all AHA isoforms (Supplemental Figure S4, E and F). As expected, immunoblots using anti-AHA1 antibodies indicated that the expression of AHA1 isoform was absent in these plants (Supplemental Figure S4G). Comparing these results with our previous findings suggests that both AHA1 and SYP132 total protein levels are altered during *Pst* DC3000 infection and are likely linked to the moderation of SYP132 density at the PM.

### The modulation of AHA1 and SYP132 densities affects their functions at the PM

**Figure 4:**
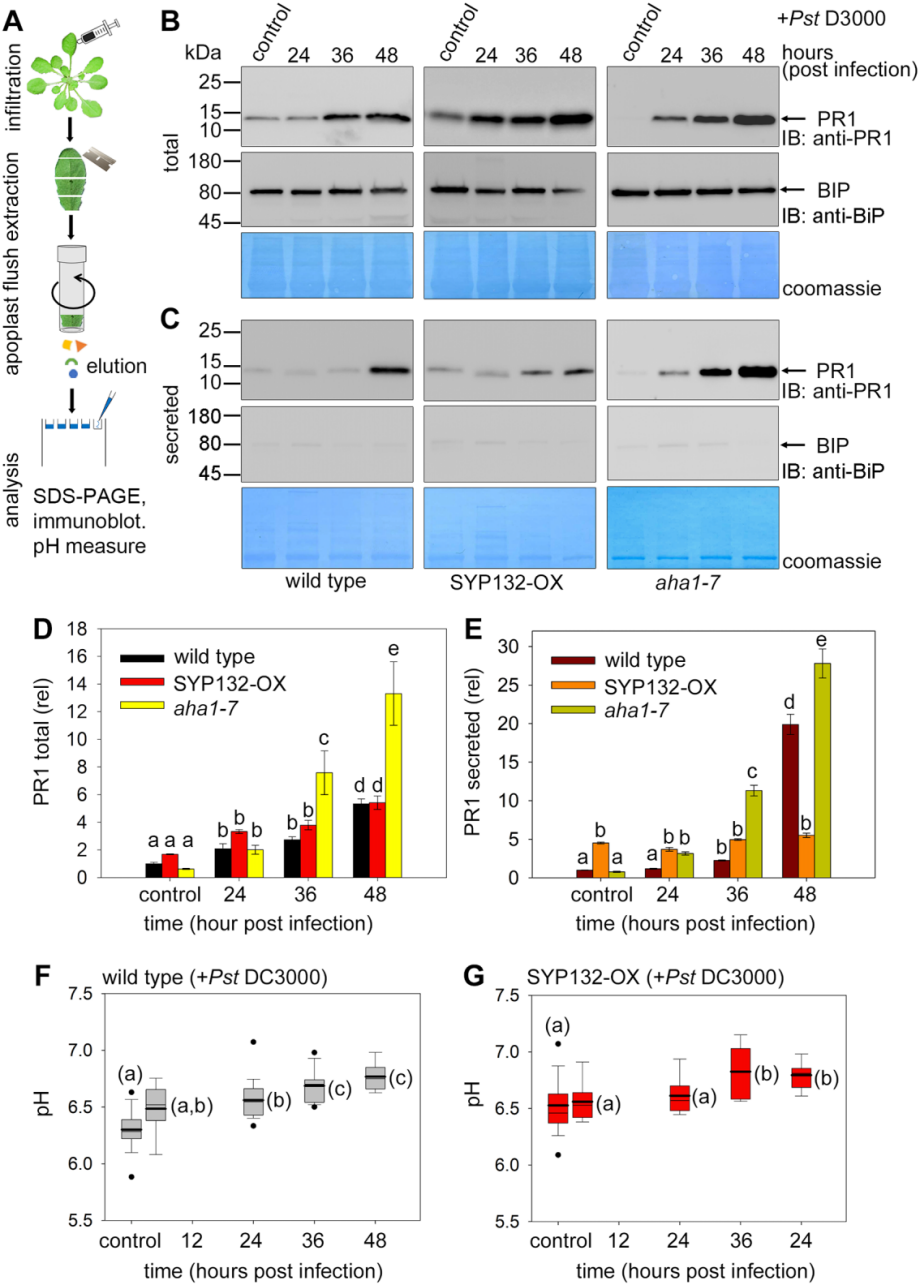
Moderation of AHA1 and SYP132 density affects pH and PR1 secretion during bacterial pathogenesis. A, Schematic for isolation and immunoblot analysis of leaf apoplast flush from Arabidopsis following infiltration with *Pseudomonas syringae* (*Pst* DC3000). B and C, Immunoblots of wild-type (left panels), p*CaMV* 35S: RFP-SYP132 Arabidopsis over-expressing SYP132 (SYP132-OX) (middle panels) and *aha1* mutant (*aha1-7* allele) (right panels) leaves at different hours postinfiltration with *Pst* DC3000 inoculum at 2.5 × 10^5^ Cfu mL^−1^ including at time 0 (control). Blots detect pathogenesis-related protein 1, PR1 at ∼16 kDa using anti-PR1 antibody (top row) and BiP at ∼74 kDa using anti-BiP antibody (middle row). BiP is an ER resident protein and its absence in apoplast-flush indicates purity of the preparations. Coomassie-stained membranes (bottom row) show total protein loading in each lane. Representative immunoblots show (B) Arabidopsis leaf lysates for detecting total protein, and (C) Leaf apoplast flush for detecting secreted proteins. To detect if any BiP was present in the apoplast flush samples, the anti-BiP immunoblot for was exposed for about 5× times longer compared to the total. Purity of apoplast flush samples was ∼99.4%, calculated using the BiP immunoblot as reference, and **Figure 4** (continued) factoring in the enhanced band intensity due to over-exposure. Black lines (left) indicate positions of molecular mass markers, and black arrows (right) indicate expected band positions. *N* = 3 with ≥10 plants for each time point per experiment. D and E, Bar graphs for PR1 total protein (D) and secreted PR1 in apoplast flush (E) calculated from immunoblots of wild-type, SYP132-OX and *aha1* mutant Arabidopsis. Data are mean ± se PR1 levels quantified from immunoblots using ImageJ software, normalized to total protein per lane detected using Coomassie stain and plotted relative to wild-type control. Statistical significance assessed using ANOVA is indicated by letters (*P* < 0.001), *N* = 3. F–G, Box plots with error bars depicting apoplast flush pH measured from wild-type (F) and SYP132-OX (G) Arabidopsis leaves at different times post *Pst* DC3000 infection including at time 0 (control). Thin horizontal lines represent the median, bold horizontal lines represent the mean, box limits show the 25th and 75th percentiles. Outliers that exceed their whisker range (1.5× interquartile range) are represented by dots. Apoplast flush collected from three leaves from each plant was pooled, and pH was measured using a micro pH electrode. Statistical significance using ANOVA is indicated by letters (*P* < 0.001). *N* = 3 with ≥5 plants for each time point per experiment.

How does SYP132 density at the plasma membrane affect secretory functions of this SNARE for plant immunity? To address this question, we tracked SYP132-mediated exocytosis of PR1 in the apoplast flush. The total cellular and secreted PR1 accumulated was detected using immunoblot (Figure 4, A–E). Purity of the apoplast flush determined using the detection of ER marker BiP as reference (Figure 4, B and C) was ∼99.4%. The band intensity for PR1 was quantified using the ImageJ software, normalized to total protein in each lane, and plotted relative to the wild-type control (Figure 4, D and E).

We observed that bacterial infection triggered increases in total PR1 in leaf tissue of wild-type, SYP132-OX, and *aha1-7* mutant Arabidopsis (Figure 4, B and D). PR1 secretion and accumulation in the apoplast was almost 20-fold higher than the control within 48 h postbacterial infection in the wild-type plants (Figure 4, C and E). In SYP132-OX plants, secreted PR1 levels were about four-fold higher compared with the wild-type plants up to 24 h postbacterial infection, but subsequently the secreted PR1 levels did not increase. Conversely in *aha1-7* mutant Arabidopsis, PR1 accumulation in the apoplast increased exponentially by 36 h postinfection and was remarkably higher by over six-fold than in wild-type and SYP132-OX plants (Figure 4, C and E). The total PR1 levels in the *aha1-7* mutant compared with the wild-type and SYP132-OX increased by two-fold (Figure 4, B and D), as can be expected due to increased PR1 accumulation in the apoplast in these plant lines. Together with the observations for SYP132 re-distribution from the plasma membrane (Figure 2), these data suggest that moderation of SYP132 abundance at the plasma membrane during pathogenesis associates with AHA1 trafficking and contributes to the regulation of PR1 secretion.

To understand the impact on PM H^+^-ATPase function, measurements of apoplast flush pH were carried out using a micro pH electrode (Xia et al., 2019). An elevation of pH at 36 h following bacterial infection was noted in wild-type plants relative to control (Figure 4F), while in the SYP132-OX plants pH was higher relative to the wild-type and it increased further relative to control at 36 h postinfection (Figure 4G). These pH change corresponded to reduced AHA1 density in the plasma membrane following bacterial infection (Figure 2). Taken together our data show that during bacterial pathogenesis AHA1 and SYP132 each affect both density and function of the other protein at the plasma membrane.

### AHA1 traffic is dictated by interactions with SYP132 through the Habc domain on the SNARE

**Figure 5:**
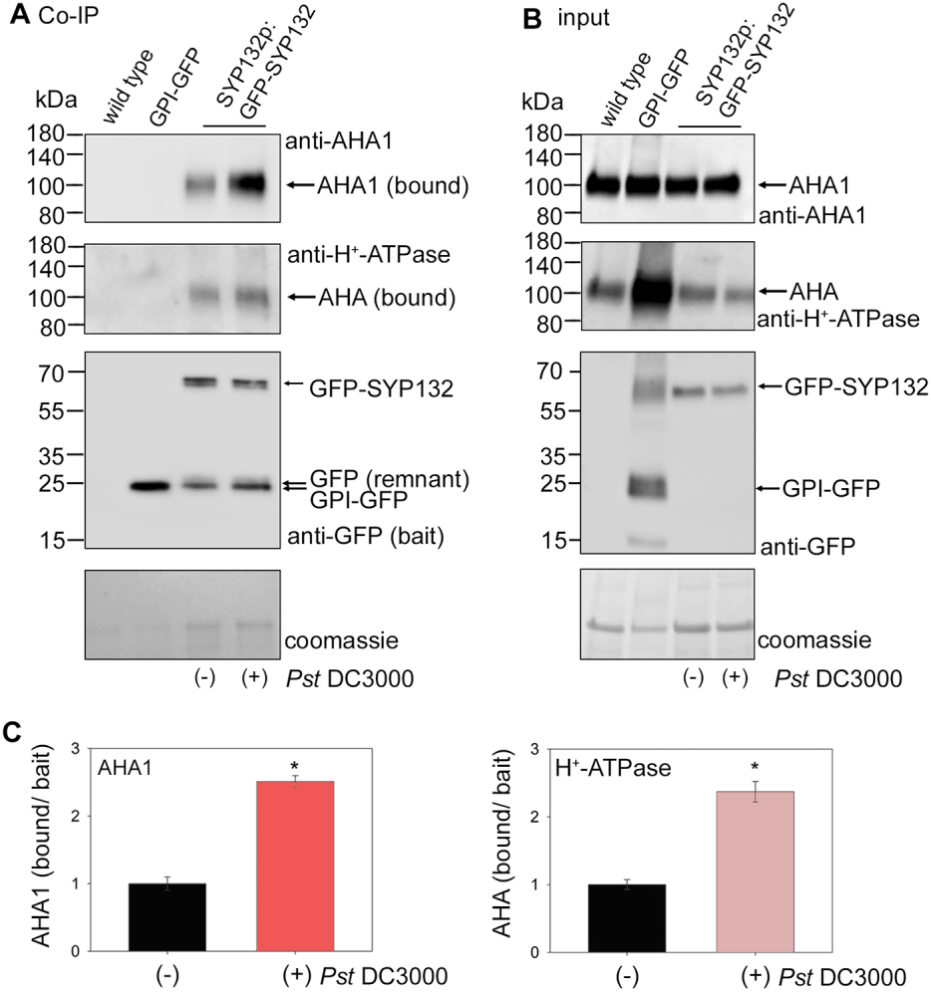
Bacterial infection influences SYP132 co-immunoprecipitant AHA1 levels. A, Immunoblots (representative) showing Co-IP assays using leaf lysates from wild-type, *CaMV* 35S: GPI-GFP or SYP132p:GFP-SYP132 Arabidopsis. SYP132p:GFP-SYP132 plants were treated over 24 h without (−) or with (+) *Pseudomonas syringae* (*Pst* DC3000) inoculum in 10 mM MgCl_2_ before lysis of leaf tissue. Input lysates were adjusted for equal GFP-tagged bait loads. Lysates were added in excess to saturate bait binding on the GFP-trap columns. Note therefore that input and bound GFP-SYP132 are similar in each case. Co-immunoprecipitants were eluted following washes to remove loosely bound proteins. Images are (top to bottom panels), immunoblots detecting AHA1 (bound) using anti-AHA1 antibodies at ∼100 kDa, all isoforms of AHA (bound) using anti-H^+^-ATPase antibodies at ∼100 kDa, baits GPI-GFP at ∼24 kDa or GFP-SYP132 at ∼62 kDa using anti-GFP antibodies and total protein per lane detected using Coomassie stain. Nonspecific AHA1 or AHA binding to the GFP-trap incubated with wild-type Arabidopsis lysate lacking GFP or with GFP-trap bound to GPI-GFP derived from 35S: GPI-GFP Arabidopsis lysates as bait was not observed, serving as experimental controls. A remnant protein at ∼24 kDa was detected using anti-GFP antibodies in the Co-IP in addition to GFP-SYP132. B, Images are (top to bottom panels), immunoblots of leaf lysates (inputs) used in the Co-IP detecting AHA1 protein using anti-AHA1 antibodies, all isoforms of AHA at ∼100 kDa using anti-H^+^-ATPase antibodies, GPI-GFP at ~24 kDa or GFP-SYP132 at ∼62 kDa using anti-SYP132 antibodies, and total protein per lane stained using Coomassie. C Mean ± se of AHA1 (left panel) and PM H^+^-ATPase (right panel) proteins bound in the Co-IP normalized to GFP-SYP132 bait. Graphs show Co-IP bound/bait following *Pst* DC3000 infection, relative to control. Data are means ± se and “*” denote statistically significant differences determined using t test (*P* < 0.001). Experiments used ≥5 plants for each, *N* = 3.

Previous studies with Arabidopsis SYP132 identified the PM H^+^-ATPase as binding partner of this SNARE (Fujiwara et al., 2014). Therefore, we analyzed if SYP132 binding with AHA1 is influenced in response to bacterial infection using in vivo co-immunoprecipitation (Co-IP) assays (Figure 5). Arabidopsis lines expressing GFP-fused SYP132 under native promoter for the SNARE (*SYP132p*:GFP-SYP132; Ichikawa et al., 2014; Xia et al., 2020) were infiltrated with *Pst* DC3000 in buffer (10 mM MgCl_2_). After 24 h postinfection, leaf tissue homogenates were prepared and applied on GFP-trap columns to capture and saturate with GFP-SYP132 as bait. As experimental control, leaf lysates from wild-type Arabidopsis lacking green fluorescent protein (GFP) protein were applied on the GFP-trap to test for nonspecific binding with the resin. In addition, leaf lysate from Arabidopsis expressing GPI-tagged GFP protein was applied to the GFP-trap to test for nonspecific interactions with the GFP protein in membranes (Figure 5, A and B). The GPI-tag is a short hydrophobic signal sequence from a glycosyl-phosphatidylinositol (GPI) protein facilitates membrane anchoring (Zhang et al., 2018). The co-immunoprecipitant proteins were eluted from the GFP-trap columns following washes to remove loosely bound proteins. Immunoblot analysis was conducted on the Co-IP eluates following resolution on SDS-PAGE to detect GFP-tagged bait proteins (GFP-SYP132 or GPI-GFP) and the bound PM H^+^-ATPase or AHA1 proteins (Figure 5A). Bait GFP-SYP132 derived from pathogen-infected Arabidopsis lysates showed almost 2.5-fold increased binding for PM H^+^-ATPase proteins and the AHA1 isoform when compared with bait derived from buffer treated plants (Figure 5, A and C). No nonspecific binding of AHA1 or any of the PM H^+^-ATPase isoforms on the GFP-trap was observed in absence of GFP-tagged bait or with GPI-GFP (Figure 5A). Thus, the data demonstrate that bacterial infection promotes SYP132 binding with AHA1.

**Figure 6:**
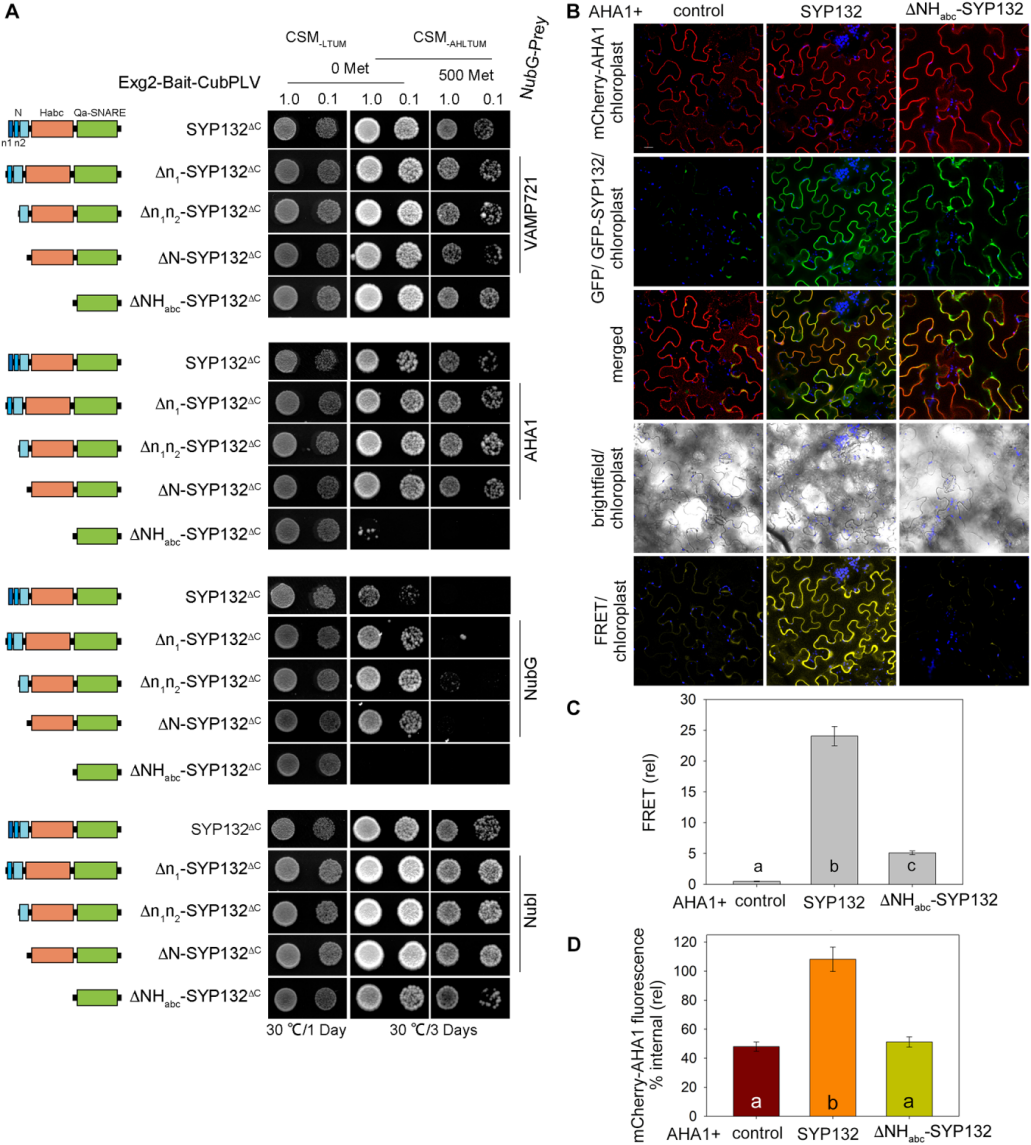
AHA1 binding with SYP132 N-terminal Habc domain on the SNARE dictates is redistribution from cell periphery. A, Yeast mating-based split-ubiquitin system with GPS to test interaction between baits (schematic on left) SYP132^ΔC (M1-Q270)^ and SYP132 truncation mutants (Δn_1_-SYP132^ΔC (R13-Q270)^, Δn_1_n_2_-SYP132^ΔC (E23-Q270)^, ΔN-SYP132^ΔC (G30-Q270)^, and ΔNH_abc_-SYP132^ΔC (E185-Q270)^) and prey proteins VAMP721 or AHA1. Bait proteins were tagged with the Exg2 GPI-signal peptide for membrane anchoring and fused with CubPLV. The prey proteins were fused with NubG. Experimental controls included bait expression with NubG (negative) and NubI (positive). Diploid yeast were dropped at OD_600_ 1.0 and 0.1 dilution and yeast growth was observed on CSM medium without Leu, Trp, Ura, and Met (CSM_-LTUM_) to verify mating and on CSM medium without Ade, His, Leu, Trp, Ura, and Met (CSM_-AHTLUM_) to identify bait–prey interactions. Addition of 500 μM Met was included to suppress bait expression as a test for the specificity of interaction. Immunoblots verifying bait and prey protein expression are shown in Supplemental Figure S5, A and B. B, Confocal images of the leaf epidermis infiltrated with water acquired on a single focal plane. *Nicotiana tabacum* epidermal cells transiently transformed using the bicistronic pFRETgc-2in1-NN vector to co-express mCherry-fused AHA1 and GFP on its own (control) or with **Figure 6** (continued) GFP-fused full length SYP132 or mutant ΔNH_abc_-SYP132. Representative confocal images are overlay with chlorophyll, showing (top-bottom) mCherry-AHA1 (acceptor reference signal, excitation at 552 nm), GFP or GFP-SNARE (donor reference signal, excitation at 488 nm), GFP or GFP-SNARE overlay with mCherry-AHA1, brightfield and mCherry-AHA1 fluorescence (FRET signal, excitation at 488 nm). Scale bar = 20 μm. *N* = 3. C, Bar graph showing FRET fluorescence signals plotted as ratios [FRET (522)/mCherry (552) relative to GFP(488)]. Fluorescence signal values were corrected for background fluorescence. Data are mean ± se of fluorescence intensity from, ≥30 cells, *N* = 3. Statistical significance by ANOVA is indicated by letters (*P* < 0.001). D, Bar graphs show mean ± se internal mCherry-AHA1 fluorescence relative to fluorescence at cell periphery as a percentage. Fluorescence measurements are from plasmolyzed cells to retract the plasma membrane and resolve the cell interior. Images were collected as Z-stacks and rendered as 3D projections (Supplemental Figure S5C) prior to analysis. Region of cell periphery, 1.5 µm width, and cell interior were traced for each cell using the brightfield image as reference. Integrated fluorescence density within the regions of interest was measured and corrected for background fluorescence (see “Materials and methods”). Statistically significant differences by ANOVA are indicated using letters (*P* < 0.001). *N* = 3. Data are from ≥6 cells per each experiment, chosen randomly for analysis. Protein expression was verified by immunoblot (Supplemental Figure S5D).

We hypothesized that binding with SYP132 might contribute to the mechanics of AHA1 trafficking. To test this theory, we set out to identify AHA1 binding domain(s) on SYP132 using the yeast GPI-signal peptide-anchored split-ubiquitin (GPS) system (Zhang et al., 2018). Previously we have analyzed SNARE interactions with its cognate SNARE partners in yeast using SYP132^ΔC^ as bait (Xia et al., 2019). So, using this established approach we tested yeast growth in cells expressing AHA1 with the full-length SYP132 or the domain truncation mutants of this SNARE as bait. Sequential deletion of the essential binding domain on SYP132 that lose AHA1 binding, we expected, would inhibit yeast growth in GPS assay. We observed that growth was not affected in yeast expressing AHA1 and the full-length SYP132 or SYP132 mutants lacking the SNARE N-terminus or its smaller subdomains (Figure 6A). However, yeast expressing AHA1 and ΔNH_abc_-SYP132^ΔC^ did not grow on media when bait protein expression was suppressed using 500 mM methionine. All the yeast expressing the cognate SNARE partner VAMP721 and SYP132 truncation mutants grew on the selection media, indicating that the mutant proteins retain cognate SNARE binding (Figure 6A). Immunoblot analysis (Supplemental Figure S5, A and B) confirmed that expression of prey and bait proteins was at similar levels in the yeast. Thus, we identified that SYP132 Habc domain is critical for AHA1 binding on SYP132.

We verified the binding between SYP132 and AHA1 and analyzed effect of these interactions on AHA1 traffic in plant cells. Imaging-based Förster resonance energy transfer (FRET) analysis was carried out using GFP-tagged SNARE as energy donor and mCherry-fused AHA1 as fluorescence energy acceptor (Zhang et al., 2019). A 2-in-1 FRET vector was used to transiently co-express mCherry-AHA1 with GFP on its own (control), with GFP-SYP132, or with the mutant GFP-ΔNH_abc_-SYP132 in *Nicotiana tabacum* (tobacco) leaf epidermis. Confocal images of transformed epidermal cells were acquired following infiltration with water (Figure 6B). The mCherry-AHA1 (acceptor) fluorescence excited by 488 nm light (donor) gave strong and highly significant signals when co-expressed with GFP-SYP132 but FRET signal was significantly lower when mCherry-AHA1 was co-expressed with GFP on its own or with GFP-ΔNH_abc_-SYP132 (Figure 6C). These data suggested that AHA1 binds with SYP132 and the interactions are lost if the NHabc domains of the SNARE are deleted.

In parallel, plasmolysis was induced in the transformed tobacco epidermal cells using 1 M NaCl (Xia et al., 2019) to resolve between the cell interior, thus facilitate quantitative analysis of mCherry-AHA1 distribution. Comparing between the full-length SYP132 and the ΔNH_abc_-SYP132 mutant which lacks AHA1 binding, we evaluated the effects of SYP132 binding on AHA1 traffic. Confocal images were acquired immediately following plasmolysis (Supplemental Figure S5C) and provided a snapshot of protein distributions in the cells using fluorescence as a reporter. Previously, using these assays, it was found that the SNARE SYP121 does not affect AHA1 resident at the cell periphery while SYP132 promoted localization of the pump proteins to the cell interior (Xia et al., 2019). mCherry-AHA1 fluorescence distribution at the cell periphery relative to cell interior was measured as before (Xia et al., 2019). Using the bright field overlay as reference, we traced to mark the cell periphery region of interest (ROI), and a second ROI was marked to cover the interior of each plasmolyzed cell. Integrated mCherry fluorescence densities across the ROIs marking the periphery and interior of each cell were measured and corrected total fluorescence for each ROI was calculated following background subtraction to determine percentage internal fluorescence relative to cell periphery. In the cells expressing mCherry-AHA1 with full-length GFP-SYP132, percent internal fluorescence for AHA1 was significantly high compared with cells co-expressing mCherry-AHA1 with GFP on its own (control) or with GFP-fused ΔNH_abc_-SYP132 (Figure 6D). These data suggest that SYP132-assisted redistribution of AHA1 to interior of the cells is blocked if the binding site on the SNARE is abolished. Immunoblot analysis (Supplemental Figure S5D) confirmed expression of the fluorophore-tagged SNAREs and AHA1. Taken together, these data demonstrate that the SYP132 Habc domain is essential for AHA1 binding and these interactions could influence AHA1 traffic from the plasma membrane.

## Discussion

The current view of the PM H^+^-ATPases is that membrane traffic alters the activity of these transporters by modulating their presence at the plasma membrane to influence plant growth, gravitropism, and abiotic stress responses (Hashimoto-Sugimoto et al., 2013; Haruta et al., 2018; Xue et al., 2018; Xia et al., 2019, 2020; Fuglsang and Palmgren, 2021). In the face of pathogen challenge, dynamic changes in the plasma membrane alter the density and distribution of several membrane proteins influencing plant immunity (Elmore et al., 2012). Our current data show that AHA1 and PR1 traffic are associated with the regulation of SNARE SYP132 density at the plasma membrane and influences bacterial pathogen proliferation in plants. We have found during bacterial pathogenesis (1) that co-ordinate trafficking pathways of AHA1 and SYP132 moderate PR1 secretion; (2) that such traffic is governed by AHA1 and SYP132 protein abundance; (3) that AHA1 interacts with SYP132, and deletion of the critical binding domain inhibits AHA1 endocytosis; and (4) that AHA1 and SYP132 each affect the density and function of the other protein at the plasma membrane. Thus, we show that the SNARE SYP132 mediates opposing trafficking pathways for PM H^+^-ATPase endocytosis and PR1 secretion during pathogenesis.

### Coordinate AHA1 and SYP132 trafficking influences their functions during pathogenesis

Plant defenses are designed to protect plants from disease by restricting microbial pathogen infection and proliferation. Stomatal defenses limit pathogen entry into the plant by closure of the stomatal pore (Melotto et al., 2006; Liu et al., 2009; Melotto et al., 2017). Immune responses including basal resistance, effector-triggered immunity as well as systemic acquired resistance hamper pathogen proliferation once in the plant tissue (Jones and Dangl, 2006). The downregulation of PM H^+^-ATPases underpins stomatal defenses to prevent pathogen entry into the plant, and also affects postinfection immune responses of the plant (Zhou et al., 2015; Kaundal et al., 2017; Melotto et al., 2017). In Arabidopsis, AHA1 and AHA2 are implicated in plant immune responses (Liu et al., 2009; Dodds and Rathjen, 2010; Zhou et al., 2015; Kaundal et al., 2017). Some of the regulation of these PM H^+^-ATPase isoforms may be connected to phosphorylation and dephosphorylation (Nuhse et al., 2007). Nevertheless, we found that bacterial infection evokes time-sensitive redistribution of AHA1 together with the SNARE SYP132 from the plasma membrane (Figure 2) influencing pH change and antimicrobial PR1 secretion to the apoplast (Figure 4). Thus, membrane traffic partakes in PM H^+^-ATPase regulation during bacterial pathogenesis.

Secretory vesicle traffic at the plasma membrane involving the SNAREs SYP121, SYP122, and SYP132 is important for plant immunity (Nuhse et al., 2003; Kwon et al., 2008; Frei dit Frey and Robatzek, 2009). For defense against fungal pathogens, SNARE SYP121 is thought to continuously recycle to and from the plasma membrane while SYP122 remains stable (Nielsen and Thordal-Christensen, 2012). Experiments using bacterial elicitors have shown that SYP122 is regulated through phosphorylation (Nuhse et al., 2003). But there is no evidence for substantial changes in the abundance of these SNAREs that might govern their function. A tight regulation of SYP132 expression is at the core of the trafficking activities mediated by this SNARE and has physiological consequences for plant growth, gravitropism, and cytokinesis (Kalde et al., 2007; Park et al., 2018; Xia et al., 2019, 2020; Fuglsang and Palmgren, 2021). Here, we observed that time-sensitive changes in SYP132 and AHA1 density associate with membrane trafficking and could impact plant health and survival (Figures 1–4 and Supplemental Figures S1, S3–S5).

There is no evidence that SYP132 over-expression suppresses secretory traffic in control conditions (Figure 4; Xia et al., 2019). Indeed, SYP132 is necessary for PR1 secretion (Figure 1, G and H; Kalde et al., 2007). We found that SYP132 expression increased during pathogenesis (Figure 3B), likely facilitating PR1 traffic at the plasma membrane. In SYP132-OX plants, where SNARE density is already elevated, secreted PR1 levels were significantly higher than in wild-type plants initially for up to 24 h post *Pst* DC3000 infection. Subsequently in time during bacterial pathogenesis, SYP132 density at the plasma membrane was moderated in these plant lines (Figure 2) and could be attributed to the consequences for PR1 accumulation in the apoplast over time (Figure 4). In SYP132-OX plants, bacterial infection promoted endocytic traffic of the SNARE (Figure 2 and Supplemental Figure S3), reduced SYP132-assisted PR1 secretion over time (Figure 4), and promoted bacterial infection. Secreted antimicrobial PR1 peptides contribute to plant defenses against bacterial pathogens (Kalde et al., 2007), so reduced PR1 secretion in SYP132-OX plants could be attributed to the increased bacterial proliferation in these lines compared with the wild-type plants (Figure 1, I–K). Thus, we suggest that SYP132 regulation is a key factor for dynamic control of AHA1 and PR1 traffic influencing plant responses against bacterial pathogens. PR1 secretion, we found, is significantly enhanced in *aha1* mutants (Figure 4) and hence the transporter appears to have a key role in co-ordinating SYP132 traffic and function at the plasma membrane. Thereby, these data also uphold previous observations that downregulation of AHA1 function effectively promotes defense against bacterial pathogens (Zhou et al., 2015; Kaundal et al., 2017).

We found that in presence of AHA1 redistribution of SYP132 from the plasma membrane moderates its density and affects PR1 secretion (Figures 2 and 4). Additional effects on immunity attributed to increased endocytosis cannot be ruled out. Even so, under normal conditions SYP132 is a low-abundant protein in plants (Enami et al., 2009). In response to bacterial infection as SYP132 levels increase, membrane traffic contributes to the redistribution of the SNARE to IM together with the AHA1 (Figures 2 and 3). SYP132 traffic is co-ordinated with AHA1 endocytosis, and it moderates secreted PR1 accumulation in the apoplast (Figure 4). Remarkably thus, our findings suggest a noncanonical mechanism that intersects the divergent AHA1 and PR1 trafficking pathways through regulation of the SYP132.

### Functional impact of AHA1 and SYP132 interactions

Together membrane traffic and transport impact immune responses in plants (Elmore and Coaker, 2011; Hashimoto-Sugimoto et al., 2013; Zhou et al., 2015; Rodrigues et al., 2017; Fuglsang and Palmgren, 2021; Klejchova et al., 2021). Our understanding of how trafficking and transport pathways are co-ordinated in plants is still developing. In recent years, the evidence for transporters and ion channels as non-SNARE binding partners in plants has suggested that such binding underpins the co-regulation of membrane traffic and transport in plant cells (Sutter et al., 2006; Honsbein et al., 2009; Karnik et al., 2013; Hachez et al., 2014; Grefen et al., 2015; Zhang et al., 2015; Karnik et al., 2017; Xue et al., 2018; Waghmare et al., 2019). Trafficking SNARE proteins, for example, bind with aquaporins plasma membrane intrinsic proteins (PIPs) to modulate their post-Golgi trafficking and fine-tune water transport (Hachez et al., 2014). At the plasma membrane the SNARE SYP121 selectively binds voltage-gated K^+^ channels KC1 and KAT1, forming higher order complexes which co-ordinate SYP121-assisted secretory traffic and K^+^ transport (Karnik et al., 2013; Grefen et al., 2015; Karnahl et al., 2018; Waghmare et al., 2019).

SNARE SYP132, however, has no influence over K^+^ channel KAT1 traffic at the plasma membrane (Xia et al., 2019). Here, we found that bacterial pathogens enhance AHA1 interactions with SYP132 (Figure 5). The Habc domain of SNARE SYP132 was identified as essential for AHA1 binding (Figure 6, A–C and Supplemental Figure S5, A and D). SYP132 truncation mutants which lack this critical AHA1 binding domain (ΔNH_abc_-SYP132) failed to facilitate AHA1 internalization from the cell periphery (Figure 6D and Supplemental Figure S5, C and D). Thus, the binding between AHA1 and SYP132 appears to be mechanistically connected to pathogen-induced endocytic traffic of the PM H^+^-ATPases.

Even so, the possibility that regulation of SYP132 during pathogenesis affecting other cargoes cannot be discounted. PM H^+^-ATPases and PIPs are both essential components of the transport machinery that energize physiological, developmental, and adaptive responses in plants including pathogen resistance (Liu et al., 2009; Rodrigues et al., 2017; Klejchova et al., 2021). At the plasma membrane, trafficking SNAREs and transporters such as PM H^+^-ATPases and PIPs are all thought to respond to bacterial elicitors influencing plant immune signaling (Vilakazi et al., 2017). Therefore, it is possible that SNARE SYP132 acts as a bridge, facilitating antimicrobial secretion and co-regulation of PM H^+^-ATPases and PIPs during pathogenesis.

The SYP132-OX plants are smaller compared to the wild-type plants (Xia et al., 2019; Figure 1D), but were more resilient to bacterial infection via the stomatal route (Figure 1, B and C). Interestingly, stomata in the SYP132-OX lines did not re-open by action of the virulent *Pst* DC3000 pathogens over time (Figure 1E), a phenotype akin to the previously observed stomatal behaviors in RPM1-INTERACTING PROTEIN4 (RIN4) knockout lines deficient in response to bacterial effectors (Liu et al., 2009; Kaundal et al., 2017). One explanation could be that stomata are always more closed or unable to open in SYP132-OX plants due to suppressed PM H^+^-ATPase function, demonstrated by Xia et al. (2019). However, it is also likely that during bacterial pathogenesis the mechanics also involve RIN4, which is a negative regulator of AHA1 activity and is thought to bind with proton pump and control its functions in stomatal immunity (Liu et al., 2009). It will be interesting, therefore, to test if SYP132 binding with AHA1 affects RIN4 binding on the PM H^+^-ATPase and if the PM H^+^-ATPase influences cognate SNARE binding on SYP132 to control vesicle traffic. Further analysis of higher-order SNARE complexes involving ion channels, transporters, and other non-SNARE binding partners is required to understand how membrane traffic and transport may be co-ordinated for immunity.

### SYP132 mediates opposing pathways for AHA1 and PR1 secretion in response to bacterial infection

While it is well-known that the plant growth hormone auxin enhances PM H^+^-ATPase activity at the plasma membrane (Hager et al., 1991), only recently has it become clear that auxin-regulated traffic of the PM H^+^-ATPases depends on the SNARE SYP132. Unusually SYP132 facilitates a redistribution of AHA1 from the plasma membrane to endomembrane compartments, thereby reducing AHA1 activity. Auxin suppresses *SYP132* expression to inhibit endocytic traffic of PM H^+^-ATPase and promote its activity (Xia et al., 2019, 2020). Complementary to these findings we know too, that the stress hormone abscisic acid suppresses H^+^-ATPase activity at the PM, facilitated by increased endocytosis of the pump together with the SYP132 (Xia et al., 2019).

Our findings put flesh to a possible mechanism by which pathogens counter host defenses. Increased auxin downregulates SYP132 expression and consequent inhibition of AHA1 endocytic traffic enhances PM H^+^-ATPase activity and promotes stomatal opening (Xia et al., 2019, 2020). Opening of the stomatal pore suppresses stomatal defenses allowing microbial pathogens to penetrate the plant (Blatt, 2000; Jezek and Blatt, 2017; Melotto et al., 2017). This model is consistent with evidence that bacterial pathogens, including the *Pseudomonas* *syringae* can enhance auxin biosynthesis in the plant leading to the inhibition of host defenses to promote pathogen infection of plant tissue (McClerklin et al., 2018). Furthermore, the SNARE SYP132 mediates secretion of antimicrobial PR1 peptides to the apoplast in response to bacterial infection (Kalde et al., 2007; Figure 1, G and H and Supplemental Figure S1H), so the downregulation of SYP132 expression by auxin would suppress PR1 secretion, inhibiting PR1-associated antimicrobial activity and host defense signaling (Jones and Dangl, 2006). Thus, our discovery of SYP132 mediating opposing traffic of AHA1 and PR1 during pathogenesis substantiates the existing knowledge for pathogen-induced auxin biosynthesis and suggests that trafficking of the SNARE itself connects both auxin- and pathogen-regulated immune responses in plants.

**Figure 7:**
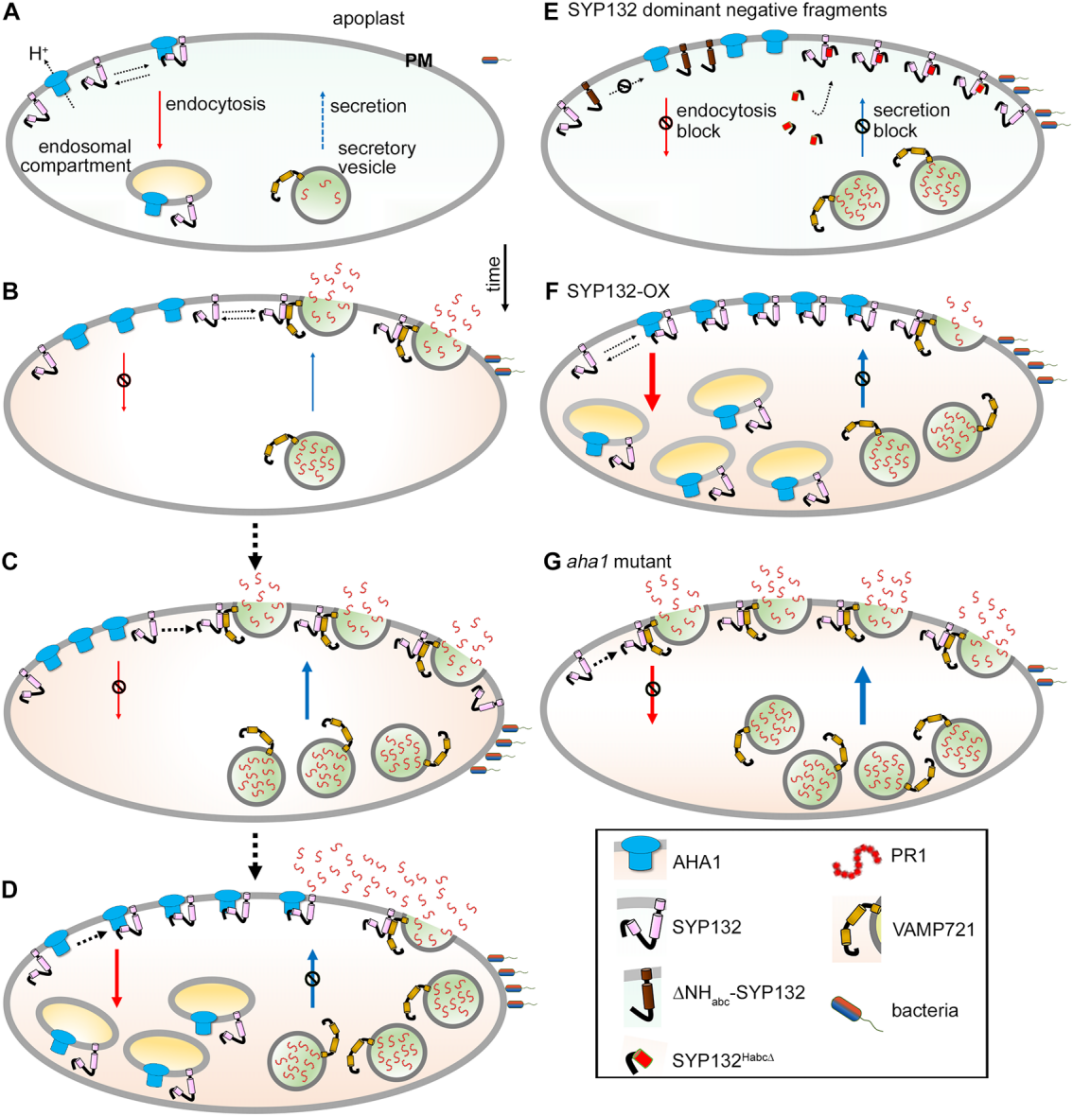
SNARE SYP132 mediates divergent trafficking pathways of PM H^+^-ATPase AHA1 and PR1 during bacterial pathogenesis. Schematic model depicting a snapshot of the events for SYP132, AHA1, and PR1 that follow pathogen infection. A, Regulation of proton (H^+^) ATPase density and function at the plasma membrane is mediated by the trafficking SNARE SYP132 which binds AHA1 and promotes its endocytosis. B–D, During bacterial pathogenesis co-ordinated SYP132 and AHA1 traffic regulates SNARE density and functions. SYP132 is diverted for antimicrobial PR1 secretion essential for pathogen defenses, and consequently AHA1 endocytosis is suppressed (B). SYP132 expression increases and supports the increasing demand for PR1 secretion (C) AHA1 abundance increases and as interactions between AHA1 and SYP132 are enhanced co-ordinate endocytic trafficking of SYP132 with AHA1 moderates SYP132 density and PR1 secretion (D). E, Over-expression of dominant-negative ΔNH_abc_-SYP132 lacking the critical binding (Habc) domain titrates out the full-length SYP132 and prevents SYP132–AHA1 interactions to block AHA1 endocytosis. The dominant-negative SYP132 peptide (SYP132^HabcΔ^) lacks the Qa-SNARE motif but retains the Habc domain that actively binds full-length SNARE proteins preventing normal SYP132 functions. Over-expression of the SYP132^HabcΔ^ therefore blocks AHA1 endocytosis and inhibits PR1 secretion. F, SYP132 overexpression (SYP132-OX) promotes both AHA1 and SYP132 internalization which blocks PR1 secretion for pathogen defense. G, In the Arabidopsis *aha1-7* mutant, AHA1-dependent SYP132 endocytosis is blocked. Consequently, more SNARE is available for secretory traffic at the plasma membrane and PR1 accumulation in the apoplast is significantly enhanced. Box (bottom right) is the key to the schematic. Thicker arrows indicate increased trafficking. Bacterial infection and the secretory vesicle localized SYP132 SNARE-complex partner VAMP721 are shown. Other secretory SNARE-complex components are not shown for simplicity. Sizes are not to scale.

In summary (Figure 7), we uncover exciting evidence suggesting that regulation of SNARE SYP132 density at the plasma membrane co-ordinates divergent AHA1 and PR1 trafficking pathways during bacterial pathogenesis. In normal conditions, SYP132 is a typically low abundant SNARE (Enami et al., 2009; Xia et al., 2019), one which auxin further suppresses to suppress AHA1 internalization and facilitate “acid” growth (Xia et al., 2019, 2020). However, it is in the nature of this SNARE to also contribute to opposing secretory traffic associated with pathogen defense (Kalde et al., 2007). Expression of the SNARE is upregulated corresponding in time to the increased demands of defense-related secretory traffic in the plant. Time-sensitive membrane traffic of SYP132 is co-ordinated with AHA1 endocytosis and it moderates abundance and function of SNARE at the plasma membrane. This model is a paradigm shift to the current perception of plasma membrane SNARE-regulation since it suggests that membrane traffic partakes in the control of SNARE function at the plasma membrane. We test our model using SYP132-OX which accelerates density-driven SNARE traffic and using the *aha1-*7 where absence of the H^+^-ATPase, disconnects SNARE traffic and the moderation of secretion. A snapshot of these macroscopic events for SYP132, AHA1, and PR1 that follow pathogen infection are depicted in Figure 7.

## Materials and methods

### Plant materials and propagation

Experiments were performed with wild-type Arabidopsis (*A.* *thaliana*) Columbia-0 ecotype and derived transgenic lines, described previously, with altered SYP132 and/or AHA1 levels, the 35S *CaMV* promoter-driven RFP-SYP132, GFP-AHA1, and GFP-AHA1:RFP-SYP132 (Hashimoto-Sugimoto et al., 2013; Cao et al., 2019; Xia et al., 2019) and the *aha1-7* null mutants (Haruta et al., 2010). Stable expression of GFP-fused with a hydrophobic signal sequence from a GPI protein as described before (Zhang et al., 2018), in wild-type Arabidopsis was 35S *CaMV* promoter-driven (35S: GPI-GFP). Arabidopsis plants were grown on half-strength Murashige and Skoog (MS) media (Sigma-Aldrich), containing 0.8% w/v agar or on soil under standard conditions of 8-h light/16-h dark, 18°C/22°C (light/dark) cycle under 150 µmol m^−2^s^−1^ light and at 60% restricted humidity (RH). Wild-type *N.* *benthamiana* and *N.* *tabacum* (tobacco) plants were grown in soil at 26°C and 70% relative humidity under a 16-h light/8-h dark cycle for 4- to 6 weeks.

### Plant transformation

*Agrobacterium tumefaciens* GV3101 harboring the genes of interest in binary 2in1 vectors, pFRETgc-2in1-NN or 35S *CaMV* promoter-driven constructs as described before (Hecker et al., 2015; Xia et al., 2019) were used for all transient and stable plant transformations. Fully expanded leaves from *N.* *benthamiana* and *N.* *tabacum* were used for transient transformation of epidermal cells as described previously (Tyrrell et al., 2007). Stable Arabidopsis transformants were generated using floral dip method and isolated using Basta or hygromycin selection as described before (Clough and Bent, 1998; Karimi et al., 2002; Xia et al., 2019).

### *Pseudomonas syringae* propagation

*Pseudomonas syringae* pv. *tomato* DC3000 (*Pst* DC3000) were cultured as described previously (Tornero and Dangl, 2001). Briefly, bacteria grown on a plate of King’s broth agar medium (King et al., 1954) with the appropriate antibiotics for 24 h at 25°C were suspended in 10 mM MgCl_2_ buffer and inoculum was prepared by diluting the bacterial suspension as required (2.5 × 10^4^ to 5 × 10^7^ Cfu mL^−1^).

### *Pseudomonas syringae* flood inoculation

As described before (Ishiga et al., 2011), 2-week-old Arabidopsis seedlings growing on plates were flood inoculated in sterile conditions by pouring 15 mL of buffer (10 mM MgCl_2_, 0.025% v/v Silwet-L77, control) or *Pst* DC3000 inoculum in buffer at 5 × 10^6^ or 5 × 10^7^ Cfu mL^−1^. Plates were decanted after 20 s, sealed, and returned to the growth room for 4 h at 24°C, under 150 µmol m^−2^ s^−1^ light. Subsequently, the flood-inoculated seedlings were transplanted to soil and grown under the standard 8-h light/16-h dark, light/dark cycle, at 18°C/22°C with 150 μmol m^−2^ s^−1^ light and 60% RH.

### *Pseudomonas syringae* infiltration

Direct infiltration of *Pst* DC3000 into leaf epidermis of 4- to 5-week-old soil-grown Arabidopsis was followed as previously described (Shen et al., 2013; Rufian et al., 2019). Briefly, leaves were poked with a needle and infiltrated abaxially with 10 μL of buffer (10 mM MgCl_2_) or *Pst* DC3000 inoculum in buffer using a needleless syringe. Pathogen infiltrated plants were returned to growth chambers with standard growth conditions for the duration of the experiment. *Pst* DC3000 inoculum at 2.5 × 10^5^ Cfu mL^−1^ was used for all experiments except for dose-dependent experiments where additional inoculum concentrations were also tested. Leaves were sampled at 24 h intervals following infiltration. To detect secreted PR1 in *N.* *benthamiana*, leaves were transformed and infected with *Pst* DC3000 as above for 48 h, prior to extraction of apoplast flush for immunoblot analysis.

### *Pseudomonas syringae* proliferation assay

To measure infection, bacteria growing in infected plants were counted. Arabidopsis leaves (infiltrated) were sampled at 24 h intervals following *Pst* DC3000 challenge. Flood-inoculated seedlings were harvested, washed thoroughly with distilled water to remove bacteria thriving on the leaf surface, and weighed. Harvested leaf tissue was immediately ground in 1 mL of distilled water using a pestle until homogeneous. The extract was serially diluted with water and 5 μL drops were applied on King’s broth agar plates with the appropriate antibiotics and incubated at 25°C. After 36 h of incubation, the number of colonies was counted on the plate for each drop that had approximately between 5 and 50 Cfu. For flood-inoculated seedlings, bacterial infection was calculated as Cfu mg^−1^ of tissue, and similarly for infiltrated leaves, bacterial colonies were calculated as Cfu leaf^−1^ and plotted on a logarithmic scale against time (Tornero and Dangl, 2001; Ishiga et al., 2011).

### Disease severity measurement from chlorotic leaves

Measurement of disease severity was as described in (Jeger and Viljanen-Rollinson, 2001). The diseased leaf area showing chlorosis/necrosis was measured using the ImageJ software and disease severity (%) was calculated using the formula:

$$\text{Disease severity }\left(\text{\%}\right)=\frac{\text{Total diseased leaf area}}{\text{Total leaf area}}\text{ x }100.$$

### Measurement of chlorophyll pigment content

Chlorophyll pigment content was measured following the protocol previously described in (Harris and Baulcombe, 2015). Briefly, excised 4 mm leaf disks were fully submerged in dimethylsulfoxide (DMSO) overnight at 4°C to extract the chlorophyll pigments. Absorbance at 665 and 647 nm was measured for the chlorophyll extract diluted 1:3 in DMSO and the chlorophyll a and b levels were calculated using the formulae:

chlorophyll a = (12 × A_665_) – (2.79 × A_647_)

chlorophyll b = (20.78 × A_647_) – (4.88 × A_665_)

Total chlorophyll pigment (a + b) content was calculated as μg mm^−2^ leaf and normalized to control to obtain % of chlorophyll reduction.

### Leaf dehydration assay

Leaf dehydration was assayed as a measure of water loss through “open” stomata reflected through a reduction in leaf weight (Hopper et al., 2014). Briefly, leaves from 5-week-old Arabidopsis were detached and placed on buffer (10 mM MgCl_2_, 0.025% Silwet-L77, control) or on *Pst* DC3000 inoculum in a closed chamber for 1 h. Each treated leaf was weighed before and after incubating at 22°C under 400 μmol m^−2^ s^−1^ PPFD light for different times. Change in leaf weight relative to control was calculated and plotted against time.

### Total RNA isolation and RT-qPCR analysis

Total RNA was extracted and purified by TRIzol Plus RNA Purification Kit (Invitrogen Waltham, MA, USA) as per the supplier’s instruction. Isolated total RNA was quantified by NanoDrop UV–Vis Spectrophotometer (Thermo Fisher Scientific, Waltham, MA, USA) before complementary DNA synthesis using QuantiTect Reverse Transcription Kit (Qiagen, Hilden, Germany). RT-qPCR reactions were performed using gene-specific primers (see Table 1), using the StepOnePlus Real-Time PCR System (Applied Biosystems, Waltham, MA, USA) and as per the recommendation of Brilliant III Ultra-Fast SYBR Green qPCR Master Mix (Agilent, Santa Clara, CA, USA). Fold change in the gene expression following *Pst* DC3000 treatment was calculated as 2^−ΔΔCT^ using reference gene, the mitochondrial 18S rRNA (AtMg01390).

**Table 1:** List of gene specific RT-qPCR primer sequences used in the study

Gene name	Gene ID	Primer (5′−3′)	Citation
SYP132	At5g08080.1	CGGAAATCCAAGAACGTCAT	Xia et al. (2019)
		TCTCCTTGTGCATCAACCAA	
PR1	At2g14610	GCTACGCAGAACAACTAAGAGG	Espunya et al. (2012)
		GCCTTCTCGCTAACCCACAT	
AHA1	At2g18960	CACAACCAAGAAAGATTACGG	Wang et al. (2012)
		CTTCTCTTGGCTTGCTCTG	
AHA2	At4g30190	CACTTCACGGTTTACAGCC	Wang et al. (2012)
		GCTTCACGACTGATTCCAC	
18S	AtMg01390	GATGAGCCTGCGTAGTATTAG	Armengot et al. (2016)
		AGTCATTCCGAAGAACACTTGC	

### Custom antibody synthesis

Polyclonal antibodies against the Arabidopsis proteins were raised in rabbits following immunization with synthetic protein-specific peptide, SYP132^peptide^ (FELPRGQSSREG) for SYP132 (Agrisera, Vännäs, Sweden) and the AHA1^peptide^ (LDIDTAGHHYTV) for the AHA1 (Eurogentec, Seraing, Belgium). To verify the specificity of antibodies, the diluted antibody suspensions were incubated with the synthetic peptides used for immunization (100 µg peptide/µL of antibody in blocking), respectively before incubating with the immunoblot membranes. Loss of protein-specific bands in immunoblots probed with peptide-saturated antibodies confirmed specificity of the antibodies in protein detection.

### Immunoblot analysis

Samples were suspended in the Laemmli buffer and resolved on SDS-PAGE using 4%–20% gradient gels (Bio-Rad, Hercules, CA, USA) at ∼5 mg/lane total protein. For immunoblot, proteins resolved on gels were transferred to nitrocellulose membranes (Bio-Rad) using Transblot (Bio-Rad). After incubating in blocking buffer (5% [w/v] nonfat dry milk, 1×Tris-buffered saline [TBS; 150 mM NaCl, 50 mM Tris–HCl, pH 7.5, 0.1% Tween-20]), membranes were first probed with primary antibodies diluted in 2.5% (w/v) nonfat dry milk, 1× TBS; anti-SYP132 (1:3,000, Agrisera), anti-AHA1 (1:3,000, Eurogentec), anti-RFP (1: 10,000; Abcam, Cambridge, UK), anti-GFP (1:10,000; Abcam), anti-H^+^-ATPase (1:10,000; Agrisera), anti-PR1 (1:10,000; Agrisera), anti-BIP (1:10,000; Agrisera), anti-HA (1:10,000; Roche, Basel, Switzerland), or anti-VP16 (1:10,000; Abcam). Subsequently, after washing with wash buffer (1× TBS), secondary antibody goat anti-rabbit-horseradish peroxidase conjugate (1:20,000; Abcam) was applied. Proteins were visualized using SuperSignal West Femto Maximum Sensitivity Substrate (Thermo Scientific) and imaged by Fusion Chemiluminescence imager (Vilber, Marne-la-Vallée, France). Total proteins were visualized by staining the membrane with Coomassie or Ponceau. Band density was measured by densitometry using the ImageJ software.

### Co-immunoprecipitation

Protein homogenates from Arabidopsis leaf tissue were diluted with the binding buffer (PBS [10 mM Phosphate, 2.68 mM KCl, 140 mM NaCl, pH 7.45], 0.01% v/v Triton X-100, 0.05 mM DTT, 0.01% CHAPS, protease inhibitor [Thermo Scientific]) for equal GFP-SYP132 levels and incubated with GFP-Trap agarose beads (Chromotek, Munich, Germany) for 1 h at 4°C with gentle shaking. Bait proteins were added in excess, to saturate the trap. The beads were collected and washed 3 times with washing buffer (binding buffer with additional 150 mM NaCl) and the bound proteins were eluted with 2× Elution buffer (50 mM Tris–HCl, pH 6.8, 5% w/v SDS, 2 mM EDTA, 0.1% v/v Triton X-100, 50 mM DTT, 12% v/v glycerol, 0.05% w/v bromophenol blue).

### Microsomal membrane isolation and fractionation

Isolation of microsomal total membranes and purification of PM and IM fractions using aqueous two-polymer phase system were performed as described previously (Xia et al., 2019). Briefly, fresh Arabidopsis leaves were ground in homogenization buffer (25 mM HEPES-KOH, pH 7.5, 330 mM sucrose, 5 mM EDTA-KOH, 0.6% [w/v] polyvinylpyrrolidone [PVP40; Sigma-Aldrich], 0.2% [w/v] BSA, 5 mM dithiothreitol [DTT]. 0.5 mM phenylmethylsulfonyl fluoride, 5 mM ascorbic acid, and protease inhibitor cocktail [Thermo Fisher Scientific]) using mortar and pestle. The resultant homogenates were filtered through Miracloth (Merck, Kenilworth, NJ, USA) before centrifugation at 8,000 *g* at 4°C for 15 min. Microsomal pellets were collected by centrifuging supernatants at 100,000 *g* at 4°C for 1 h and resuspending in resuspension buffer (buffer R: 5 mM potassium phosphate [a mixture of 200 mM KH_2_PO_4_ and 200 mM K_2_HPO, pH 7.8], 330 mM sucrose, 0.1 mM EGTA, 0.1 mM EDTA-KOH, 100 mM phenylmethylsulfonyl fluoride, 1 mM DTT, and protease inhibitor cocktail. Microsomal membranes were further separated by adding them to a 27 g phase buffer (6.5% [w/v] PEG3350, 6.5% [w/v] Dextran T500, 330 mM sucrose, 5 mM potassium phosphate, and 3 mM KCl). The protein quantity in microsomal fractions was determined using Bradford (Bio-Rad) with BSA Fraction V as a standard. Upper and lower phases were collected separately by centrifugation at 1,500 *g* at 4°C for 15 min. The upper and lower phases were repartitioned twice with fresh clear lower and upper phases buffers to enhance purity of the preparations. Finally, purified phases were diluted with buffer R three-fold (upper phases) or 10-fold (lower phases), and plasma (upper) and internal (lower) membranes were collected by centrifugation at 100,000 *g* at 4°C for 1 h and resuspended in buffer R. The membrane fraction sets were each adjusted for equal total protein relative to the respective control, and total protein load in lane was additionally verified by Coomassie staining of the immunoblot membranes.

### Confocal imaging

Confocal images of *N.* *tabacum leaf* epidermis were acquired as described before (Xia et al., 2019), using a Leica TCS SP8-SMD confocal microscope with spectral GaAsP detectors. Images were collected as z-stacks using 63×/0.75 NA oil-immersion objective lens (stomata) or on a single focal plane using 20× objective lens. GFP fluorescence was excited with continuous 488 nm light and collected over 500–535 nm. RFP was excited with 552 nm light, and RFP fluorescence emission was collected over 560–615 nm. Chloroplasts in leaf tissue were visualized by exciting chlorophyll a with 488-nm light and fluorescence emission was collected over 650–670 nm. FRET analysis was carried out as described in (Zhang et al., 2019) following confocal imaging of the leaf epidermal cells transiently transformed to express pFRETgc-2in1-NN (Hecker et al., 2015) constructs harboring GFP and mCherry protein fusions. The GFP fluorescence (donor) was excited with 488-nm light and signals were collected over 500-535 nm and the FRET signal was collected over 590-645 nm. Separately, the mCherry fluorescence (acceptor) emission was also collected over 590–645 nm with excitation of 552 nm light. After subtracting background, the changes in FRET were calculated as the FRET/mCherry fluorescence ratio and normalized to the GFP signal for expression correction. Bleed-through from donor and acceptor fluorescence was corrected based on the respective fluorophore signals, and their absorption and emission spectra.

### Quantification of fluorescence distribution

Quantification of cell internal–peripheral fluorescence distribution from confocal images of transformed *N.* *tabacum* leaf epidermis, or guard cells in Arabidopsis leaves was as described previously (Marwaha and Sharma, 2017; Xia et al., 2019). Briefly, confocal images were collected as Z-stacks and rendered as 3D projections prior to analysis. For each cell, ROIs demarcating cell periphery (width of 1.5 μm) and cell interior were demarcated for each cell using the bright-field image for reference. Integrated fluorescence density was measured using the ImageJ software for each ROI and corrected for background fluorescence using the equation [corrected total fluorescence = integrated density – (area of selected ROI × mean fluorescence of background)]. The proportion of fluorophore distribution at the cell periphery and interior was represented as bar graphs of mean internal/peripheral fluorescence ratios.

### Apoplast flush extraction

Apoplast flush containing pool of proteins secreted out of the cell was collected from intact leaves as described in Xia et al. (2019). Briefly, *Pst* DC3000 or buffer treated rosette leaves were infiltrated with wash buffer (10 mM MES-KOH, 10 mM MgCl_2_, pH 5.6, protease inhibitor [Thermo Scientific]), and immediately cut into 5–6 mm wide strips, stacked, rolled, and loaded vertically (cut ends top and bottom) in Proteus 1-step Batch Mini Spin Column (Neo Biotech, Pasadena, CA, USA). Apoplast fluid was flushed out by centrifugation at 10,000 *g* and 4°C for 1 min. Fluid from ∼15 different leaves from 10 to 12 plants was pooled. In parallel, whole leaf lysates (total) were prepared in homogenization buffer (see above). Total protein was quantified using Bradford assay (Bio-Rad) and adjusted for equal loading on gels.

### SUS yeast growth assays with the GPS system

Yeast mating-based split-ubiquitin (SUS) assays using the GPI Signal Peptide-Anchored Split-Ubiquitin (GPS) system were performed by expressing bait proteins in the pEXG2Met-Dest vector and prey proteins in the pNX35-Dest vector (Zhang et al., 2018) and as described before in Xia et al., 2019). Briefly, *Saccharomyces cerevisiae* (yeast) strains, THY.AP4 and THY.AP5 described before (Obrdlik et al., 2004) were transformed with bait and prey constructs, respectively. Pools of 10–15 yeast colonies were selected and inoculated into selective medium (CSM_-LM_ for THY.AP4 and CSM_-TUM_ for THY.AP5) for overnight growth at 180 rpm and 30°C. Liquid cultures were harvested and resuspended in Yeast Extract-Peptone-Dextrose (YPD) medium. Aliquots of 5 µL mated yeast were dropped on YPD plates and incubated at 30°C overnight. Then, colonies were transferred from YPD plates onto CSM_-LTUM_ plates and incubated at 30°C overnight. Diploid colonies were selected and inoculated in liquid CSM_-LTUM_ medium and grown at 180 rpm and 30°C overnight. After that, cultures were harvested and resuspended in sterile distilled water. Serial dilutions at OD_600_ of 1.0 and 0.1 in water were dropped on CSM_-AHLTUM_ plates (5 µL per spot), with addition of 500 µM Methionine to test the specific interaction at lower bait expression. Plates were incubated at 30°C, and yeast growth was imaged after 72 h. Diploid yeast cells were also dropped on CSM_-LTUM_ control plates to confirm mating efficiency and cell density, and spots were imaged after 24 h at 30°C. To verify expression, diploid yeast cells were harvested and extracted for immunoblots analysis using anti-HA and anti-VP16 antibodies to detect prey and bait proteins, respectively.

### Statistics

Data are means ± se of *N* ≥ 3 independent experiments. Statistical analysis was performed using Sigma plot 12.5 (Systat Software). Student’s t test or ANOVA with the Holm–Sidak method was used to determine significance for pairwise or multiple comparisons, respectively. For bacterial growth assays Wilcoxon–Mann–Whitney statistical test was used for comparing between two conditions, and Kruskal–Wallis statistical test was used to analyze differences between individual data points.

## Accession numbers

Transgenic lines used in the study are for AHA1 (*At2g18960*) T-DNA mutant (SALK_065288), *aha1-7* allele. Sequence data from this article can be found in Arabidopsis Genome Initiative or GenBank/EMBL databases under the following identifiers: SYP132 (At5g08080), VAMP721 (At2g25340), AHA1 (At2g18960), and PR1 (At2g14610).

## Supplemental data

The following materials are available in the online version of this article.

**Supplemental Figure S1.** Bacterial proliferation in Arabidopsis is inoculum dose-dependent and evokes SYP132-dependent antimicrobial secretion.

**Supplemental Figure S2.** Verification of custom synthesized anti-SYP132 and anti-AHA1 antibodies.

**Supplemental Figure S3.** Coordinate redistribution of AHA1 and SYP132 from the cell periphery is enhanced in response to bacterial infection.

**Supplemental Figure S4.** Verifying SYP132 and AHA1 protein expression.

**Supplemental Figure S5.** Verifying SYP132 and AHA1 protein expression and cellular distribution.

## Supplementary Material

kiac149_Supplementary_Data

## Notes

R.K. conceived research plans and designed the experiments. R.K. and L.X. prepared constructs. L.X. performed RT-qPCR and carried out yeast interaction assays. G.B. conducted pathogen infection assays. L.X., G.B., and R.K. did plant propagation and transgenic line preparation, confocal imaging, and secretion assays. G.B., L.X., and S.W. conducted biochemistry experiments. All authors contributed to data analysis. R.K. and S.W. wrote the manuscript.

The author responsible for distribution of materials integral to the findings presented in this article in accordance with the policy described in the Instructions for Authors (https://academic.oup.com/plphys/pages/general-instructions) is: Rucha Karnik (rucha.karnik@glasgow.ac.uk).
