# Supplementary material for: SNARE SYP132 mediates divergent traffic of plasma membrane H+-ATPase AHA1 and antimicrobial PR1 during bacterial pathogenesis
Source: Plant Physiol. 2022 Mar 28;189(3):1639–61. doi: 10.1093/plphys/kiac149 (PMC9237740; doi:10.1093/plphys/kiac149)
Supplement: kiac149_Supplementary_Data [file kiac149_supplementary_data.pdf]

Supplemental Figures

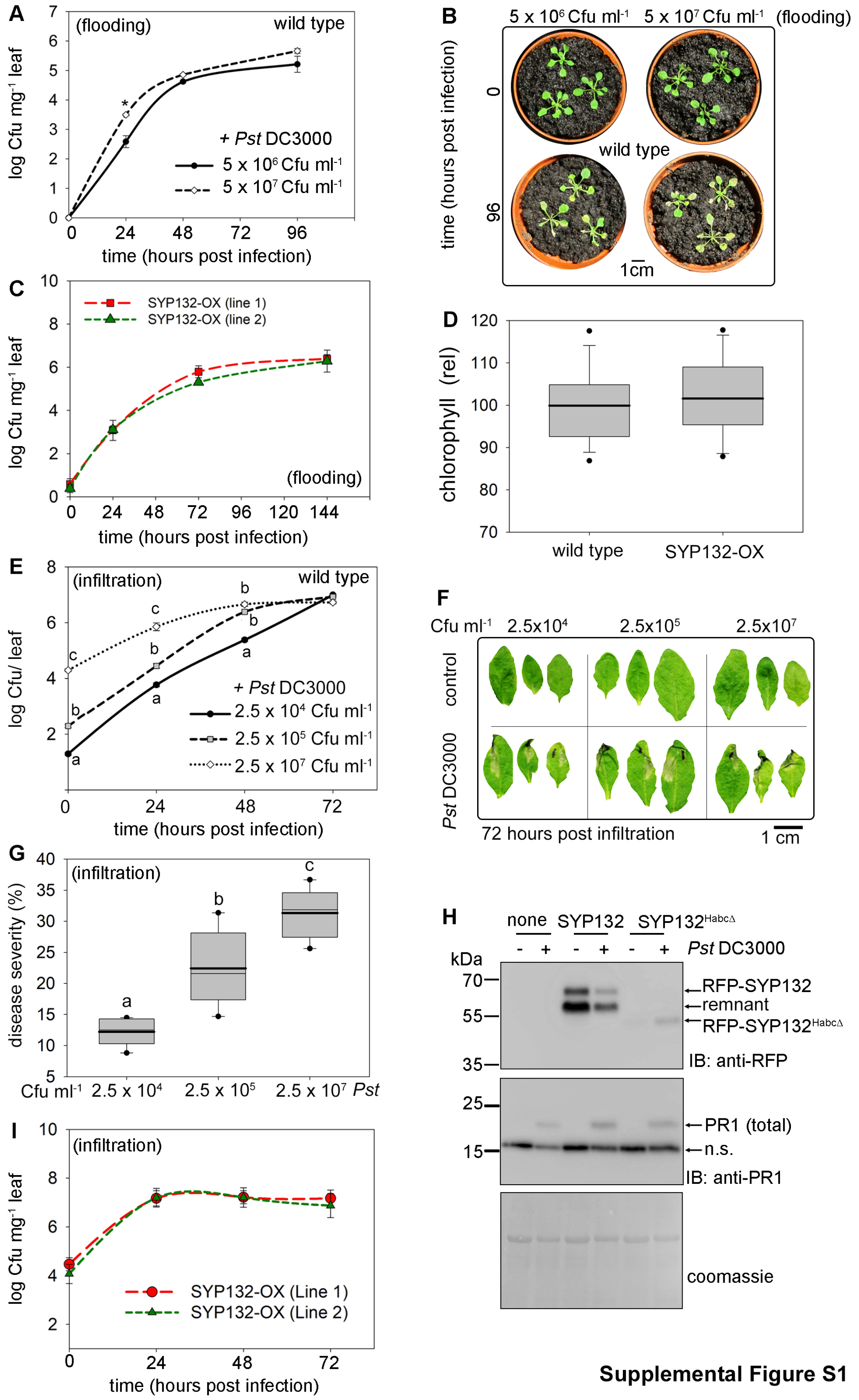

Supplemental Figure S1

Supplemental Figure S1. Bacterial proliferation in Arabidopsis is inoculum dose-dependent and evokes SYP132-dependent antimicrobial secretion.

(A) Bacterial pathogen proliferation in wild type Arabidopsis flood-inoculated using *Pseudomonas syringae* (*Pst* DC3000) inoculum at 5 x 10^6 or 5 x 10^7 Cfu ml^{-1}. Bacterial population was determined in leaves sampled at 24-hour intervals and plotted as mean ± SE colony-forming units of bacteria per mg of leaf tissue (Cfu mg^{-1}) on a logarithmic scale against time (hours post infection). Statistically significant differences assessed using Mann–Whitney–Wilcoxon test are indicated with '*' after comparing bacterial populations for different inoculum concentrations at each timepoint. ($P = 0.001$), N ≥ 3. Data are from ≥ 4 plants for each time point per experiment.

(B) Representative photographs of wild type Arabidopsis seedlings flood-inoculated using *Pst* DC3000 inoculum at 5 x 10^6 or 5 x 10^7 Cfu ml^{-1} and grown on soil for 96 hours post infection. Images are aligned digitally for comparisons. N = 3. Scale bar = 1 cm (accurate for each image).

(C) Mean ± SE colony-forming units per mg of leaf tissue (Cfu mg^{-1}) in two independently transformed p*CaMV* 35S: RFP-SYP132 Arabidopsis over-expressing SYP132 [SYP132-OX: Line1 and Line 2], flood-inoculated with *Pst* DC3000 inoculum at 5 x 10^7 Cfu ml^{-1}. Bacterial population was determined in leaves sampled at 24-hour intervals and plotted as mean ± SE colony-forming units of bacteria per mg of leaf tissue (Cfu mg^{-1}) on a logarithmic scale against time (hours post infection). Bacterial populations for different Arabidopsis lines were compared for each timepoint. Statistically significant differences were assessed using Mann–Whitney–Wilcoxon test ($P < 0.001$), N ≥ 3. Data are from ≥ 4 plants for each time point per experiment.

(D) Box plots with error bars depicting chlorophyll pigments in wild type and SYP132-OX Arabidopsis leaf tissue, calculated relative wild type. Thin horizontal lines represent the median, bold horizontal lines represent the mean, box limits show the 25th and 75th percentiles. Outliers that exceed their whisker range (1.5x interquartile range) are represented by dots. Statistically significant differences assessed using Mann–Whitney–Wilcoxon test are indicated with '*' ($P < 0.001$), N = 3, using ≥ 6 plants per experiment. Note: horizontal lines for mean and median are overlapped.

(E) Bacteria *Pst* DC3000 multiplication in wild type Arabidopsis leaves following infiltration with 10 µl *Pst* DC3000 inoculum at 2.5 x 10^4, 2.5 x 10^5 or 2.5 x 10^7 Cfu ml^{-1} in buffer measured at 24-hour intervals for 72 hours post infection. Graphs are mean ± SE *Pst* DC3000 colony-forming units (Cfu) per leaf plotted are plotted on a logarithmic scale against time (hours post-infection). Letters indicate statistically

significant differences assessed using Kruskal-Wallis test for each time point (*P* < 0.001), N ≥ 3. Data are from ≥ 4 plants for each time point per experiment.

(F) Photographs of wild type Arabidopsis infiltrated with 10 µl *Pst* DC3000 inoculum at 2.5 x 10^4, 2.5 x 10^5 or 2.5 x 10^7 Cfu ml^{-1} or buffer (10 mM $MgCl_2$, control) after 72 hours. Images are aligned digitally for comparisons. N = 3. Scale bar = 1 cm (accurate for each image).

(G) Box plot with error bars depicting % disease severity in wild type Arabidopsis infiltrated with 10 µl *Pst* DC3000 inoculum at 2.5 x 10^4, 2.5 x 10^5 or 2.5 x 10^7 Cfu ml^{-1}. Area of leaf showing necrosis was measured from leaf photographs acquired 72 hours post infiltration. Thin horizontal lines represent the median, bold horizontal lines represent the mean, box limits show the 25th and 75th percentiles. Outliers that exceed their whisker range (1.5x interquartile range) are represented by dots. Letters indicate statistical significance using ANOVA (*P* < 0.01), N = 3. Data are from ≥ 4 plants for each time point per experiment.

(H) Immunoblots (representative) of *Nicotiana benthamiana* leaf tissue, untransformed (none) or expressing full length SYP132 (SYP132) or dominant negative, the so-called Sp3-fragment ($SYP132^{Habc\Delta}$), treated with buffer (control, -) or *Pst* DC3000 (+) for 48 hours. Blots show RFP-SYP132 at approx. 61 kDa or RFP-$SYP132^{Habc\Delta}$ at approx. 49 kDa using RFP antibodies (top panel) and total *Nicotiana benthamiana* PR1 at approx. 20 kDa using anti-PR1 antibody (middle panel). Coomassie stained membrane (bottom panel, pseudo coloured) represents total protein per lane. Black lines (left) indicate position of molecular mass markers, and black arrows (right) indicate expected band positions. Additional lower molecular weight bands detected in anti-RFP immunoblots (indicated as remnant) could include cleaved or part-synthesised proteins following transformations.

(I) *Pst* DC3000 population in two independently transformed p*CaMV* 35S: RFP-SYP132 Arabidopsis lines over-expressing SYP132 (SYP132-OX: Line1 and Line2) following infiltration with *Pst* DC3000 inoculum at 2.5 x 10^5 Cfu ml^{-1}. Graphs are mean ± SE *Pst* DC3000 colony-forming units (Cfu) per leaf plotted on a logarithmic scale against time (hours) post infection. Statistically significant differences were assessed using Mann–Whitney–Wilcoxon test after comparing bacterial populations for different Arabidopsis lines at each timepoint (*P* < 0.001), N = 3. Data are from ≥ 4 plants for each time point per experiment.

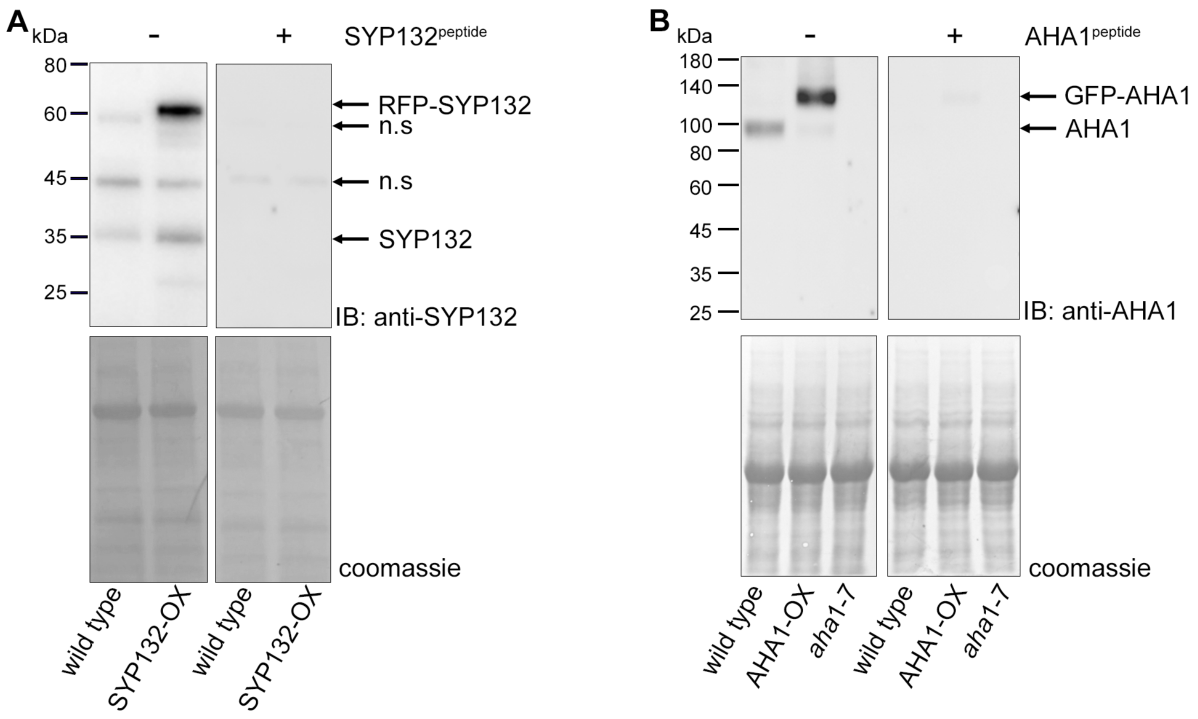

Supplemental Figure S2

Supplemental Figure S2. Verification of custom synthesized anti-SYP132 and anti-AHA1 antibodies.

Immunoblot analysis to determine specificity of custom-synthesised antibodies. Proteins from *Arabidopsis thaliana* leaf lysates were resolved on SDS-PAGE and treated with the custom synthesised antibodies incubated without (-) or with (+) the protein specific peptides with the expectation that protein-specific bands would not be detected if the antibody was saturated with the antigen prior to immunoblot. Blotted membranes were stained with Coomassie blue (lower panels) show total protein per lane. Black lines (left) indicate positions of molecular mass markers, and black arrows (right) indicate expected band positions. In each case, protein-specific bands were observed in lysates from wild type and over-expressor plants, but these bands were absent in mutant plants or if antibodies were saturated with antigen peptides prior to immunoblot.

(A) Immunoblot verifying anti-SYP132 antibodies using leaf lysates from wild type and p*CaMV* 35S: RFP-SYP132 Arabidopsis over-expressing SYP132 (SYP132-OX) Arabidopsis (Xia et al., 2019). Bands for native SYP132 at approx. 35 kDa and RFP-SYP132 at approx. 61 kDa were detected in the immunoblot analysis. Note: Non-specific (n.s.) bands detected are indicated.

(B) Immunoblot to test anti-AHA1 antibodies using leaf lysates from wild type, AHA1 over-expressor p*CaMV* 35S: GFP-AHA1 (AHA1-OX) and the *aha1* mutant (*aha1-7*) (Haruta and Sussman, 2012) Arabidopsis. Bands for native AHA1 at approx. 100 kDa and for GFP-tagged AHA1 at approx. 130 kDa were detected in the immunoblot analysis.

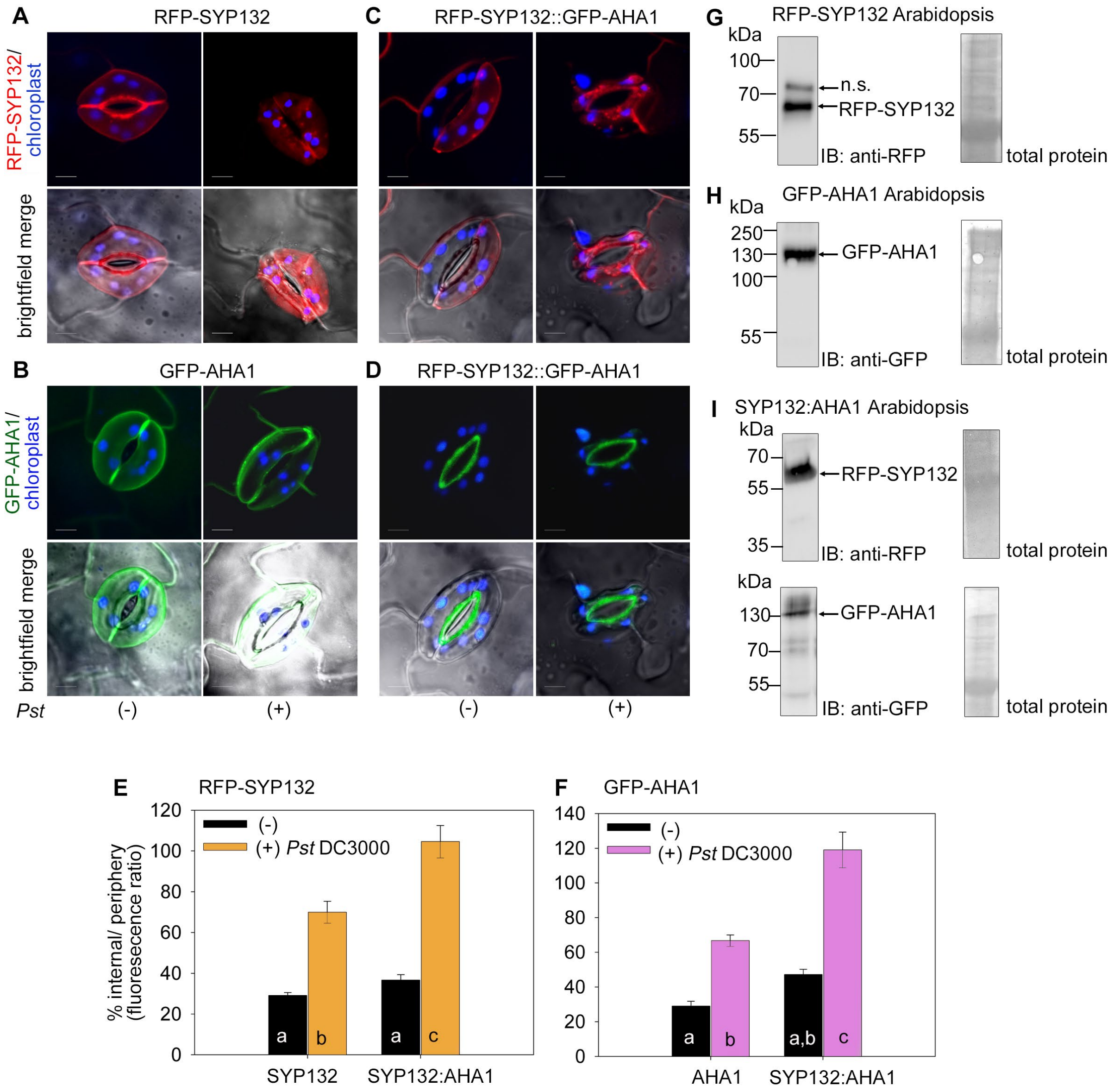

Supplemental Figure 3

Supplemental Figure S3. Co-ordinate redistribution of AHA1 and SYP132 from the cell periphery is enhanced in response to bacterial infection.

Distribution of fluorophore tagged-SYP132 and AHA1 in stomatal guard cells in four-week-old transgenic Arabidopsis infiltrated with buffer (10 mM $MgCl_2$, control, left panels) without (-) or with *Pst* DC3000 (+) inoculum at 2.5 x 10^5 Cfu ml^{-1} (right panels). Confocal images were acquired as Z-stacks and rendered as 3D projections 48 hours post-infection. Scale bar = 5 µm. Stomata for imaging were chosen at random, from two different plants for each treatment per experiment.

(A) Representative confocal images from p*CaMV* 35S: RFP-SYP132 Arabidopsis over-expressing RFP-SYP132 (SYP132-OX) detecting RFP-SYP132 (red) and

chloroplast (blue) fluorescence without (top panels) and with bright field overlay (bottom panels).

(B) Representative confocal images from p*CaMV* 35S: GFP-AHA1 Arabidopsis over-expressing GFP-AHA1, detecting GFP-AHA1 (green) and chloroplast (blue) fluorescence without (top panels) and with (bottom panels) bright field overlay.

(C-D) Representative confocal images from p*CaMV* 35S: RFP-SYP132:GFP-AHA1 plants co-expressing RFP-SYP132 and GFP-AHA1, detecting (C) RFP-SYP132 (red) / chloroplast (blue) fluorescence without (top panels) and with bright field overlay (bottom panels), and detecting (D) GFP-AHA1 (green) / chloroplast (blue) fluorescence without (top panels) and with bright field overlay (bottom panels).

(E-F) Graphs showing mean ± SE internal / periphery fluorescence ratios following background subtraction, in each guard cell for RFP-SYP132 (E) and GFP-AHA1 (F) from ≥ 12 stomata. The region of the cell periphery, 0.5 µm in width, and the cell interior were traced for each guard cell using the bright-field image as a reference. Integrated fluorescence density within the ROIs was measured and corrected for background fluorescence (see “Materials and Methods”). Letters indicate statistically significant differences determined using ANOVA ($P < 0.001$), N = 3. Data are acquired from ≥ 18 guard cells.

(G) Immunoblots (left panel), representative, using anti-RFP antibody detecting expression of RFP-SYP132 at approx. 61 kDa. Note, that a non-specific (n.s.) at approx. 80 kDa was observed.

(H) Immunoblots (left panel), representative, using anti-GFP antibody detecting expression of GFP-AHA1 at approx. 130 kDa.

(I) Immunoblots, representative, detecting expression of RFP-SYP132 at approx. 61 kDa) using anti-RFP antibody (top panels), and GFP-AHA1 at approx. 130 kDa using anti-GFP antibody (bottom panels).

Black lines (left) indicate positions of molecular mass markers, and black arrows (right) indicate expected band positions. Blotted membrane stained with Coomassie blue (right panels) show total protein per lane (G-I).

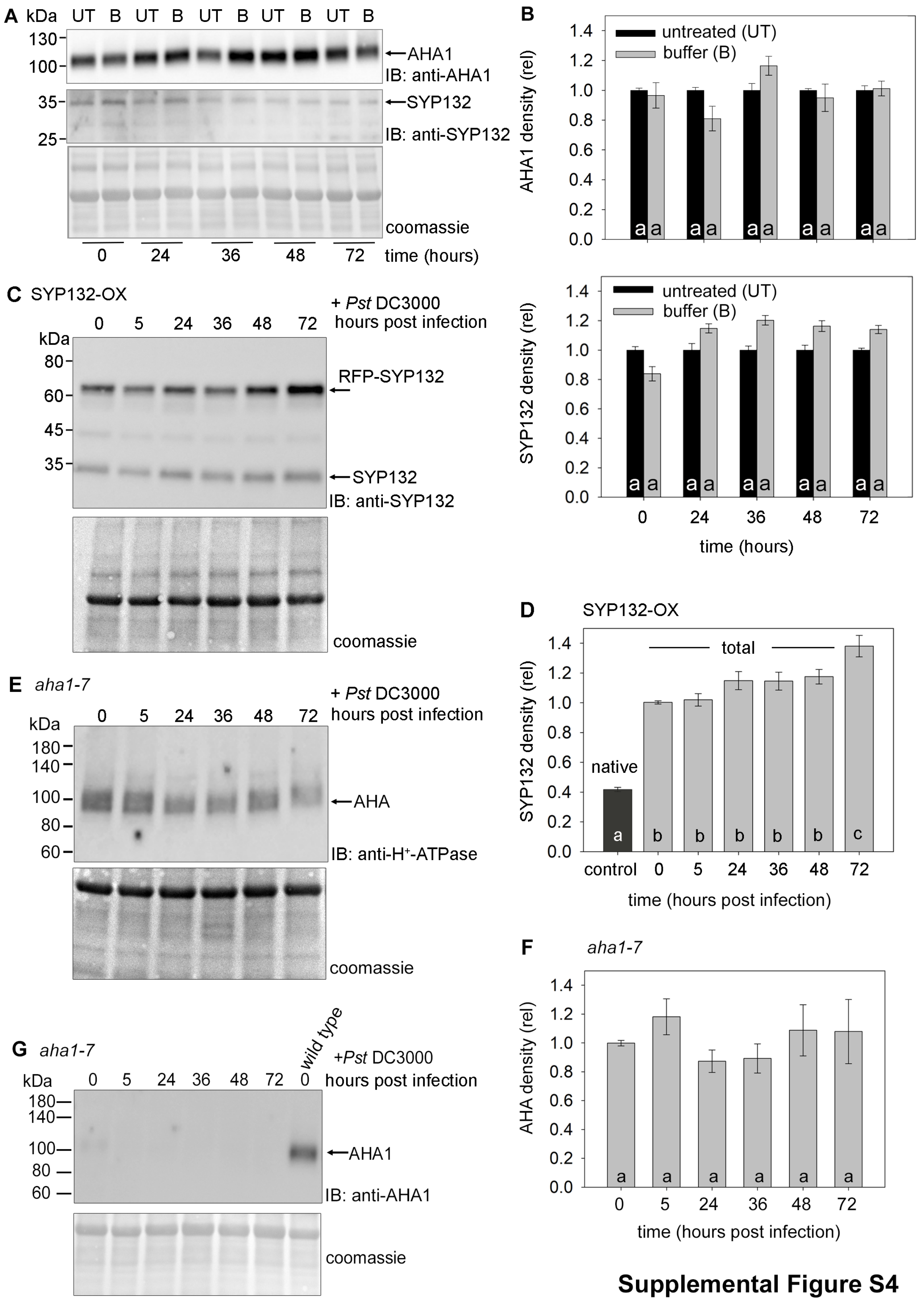

Supplemental Figure S4

Supplemental Figure S4. Verifying SYP132 and AHA1 protein expression.

(A) Immunoblots of microsomal membranes from wild type Arabidopsis untreated (UT) or mock-infiltrated with 10 µl 10 mM $MgCl_2$ buffer (B) sampled at 24-hour intervals up

to 72 hours. Native AHA1 (top panel) at approx. 100 kDa was detected using anti-AHA1 antibody. Native SYP132 (middle panel) at approx. 35 kDa was detected using anti-SYP132 antibodies. Coomassie stained membrane (bottom panel) shows total protein loaded per lane.

(B) Graphs showing mean ± SE protein levels for AHA1 (top panel) and SYP132 (bottom panel) quantified using densitometric analysis of immunoblot bands in the ImageJ software. Band densities were normalized to total protein per lane detected using Coomassie stain and plotted as relative to time zero. Letters denote statistically significant differences assessed using ANOVA ($P < 0.001$), N = 3.

(C,E,G) Representative immunoblots of microsomes purified from Arabidopsis leaf tissue harvested at 24-hour intervals for 72 hours post infection with *Pseudomonas syringae* (*Pst* DC3000) and resolved on SDS-PAGE are shown (top panels). In p*CaMV* 35S: RFP-SYP132 Arabidopsis over-expressing SYP132 (SYP132-OX) both native SYP132 at approx. 35 kDa and RFP-SYP132 at approx. 61 kDa are detected using anti-SYP132 antibodies (C), in Arabidopsis *aha1-7* mutant total AHA population is detected using anti-H^+-ATPase antibodies which detect all AHA isoforms (E), while AHA1 is detected using anti-AHA1 antibodies (G), at approx.100 kDa. AHA1 bands are detected in wild type Arabidopsis but absent in *aha1-7* mutant plants. N = 3, with $n \geq 4$ plants per time point in each experiment. Coomassie stained membranes (bottom) panels show total protein per lane. Note: Coomassie images in (C) and (E) correspond with Figures 3 (H) and (J) respectively, and therefore are the same.

(D, F) Protein levels for SYP132 (D) and AHA (F) in SYP132-OX and *aha1-7* mutant Arabidopsis respectively at different times post infection. Data are means ± SE protein levels, derived from densitometry of immunoblot bands using ImageJ software, normalized to total protein per lane detected using Coomassie stain. SYP132 density in SYP132-OX plants (D) shows native SNARE levels at time zero (black bar) and total SYP132 levels (native SYP132 + RFP-SYP132, grey bars) relative to time zero at different time intervals post infection. Letters denote statistically significant differences assessed using ANOVA ($P < 0.001$), N = 3.

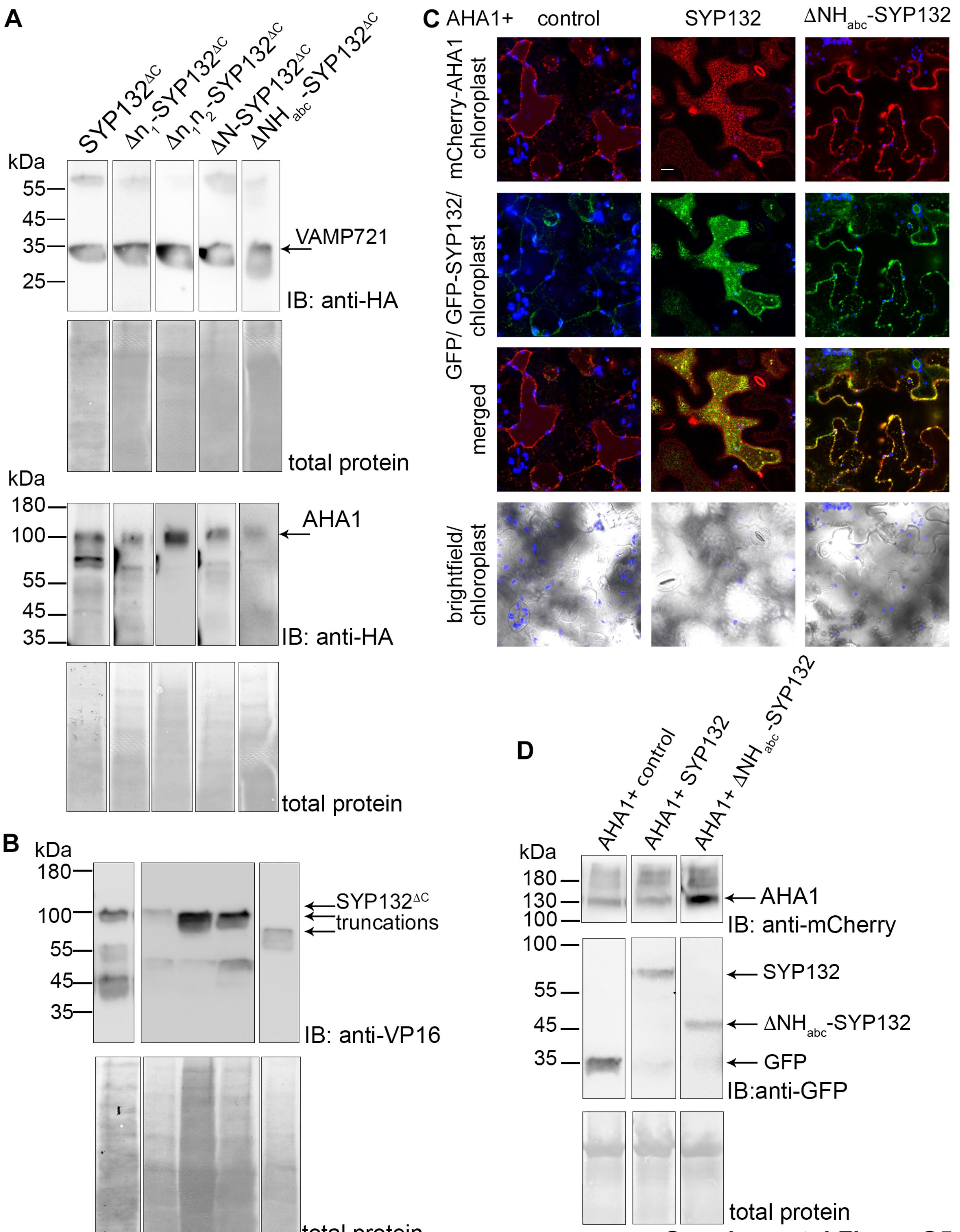

Supplemental Figure S5

Supplemental Figure S5. Verifying SYP132 and AHA1 protein expression and cellular distribution.

(A-B) Representative immunoblots detecting expression of proteins in haploid yeast used in GPS assay in (Figure 6A) resolved on SDS-PAGE to detect the expression of NubG-fused prey (A) and CubPLV fused Exg2-bait (B) proteins using anti-HA and anti-

VP16 antibodies respectively. Immunoblots detecting expression of prey proteins, VAMP721 at approx. 32 kDa and AHA1 at approx. 119 kDa, and bait proteins SYP132$^{\Delta C\ (M1\text{-}Q270)}$ at approx. 91 kDa, Δn_1-SYP132$^{\Delta C\ (R13\text{-}Q270)}$ at approx. 89 kDa, Δn_1n_2-SYP132$^{\Delta C\ (E23\text{-}Q270)}$ at approx. 88 kDa, ΔN-SYP132$^{\Delta C\ (G30\text{-}Q270)}$ at approx. 87 kDa and ΔNH_{abc}-SYP132$^{\Delta C\ (E185\text{-}Q270)}$ at approx. 70 kDa. Black lines (left) indicate positions of molecular mass markers, and black arrows (right) indicate expected band positions.

(C) Confocal images of *Nicotiana tabacum* tobacco epidermis transiently transformed with the bicistronic pFRETgc-2in1-NN vector to co-express mCherry-fused AHA1 with GFP on its own (control), with GFP-fused full-length SYP132 or the ΔNH_{abc}-SYP132, for analysis of their periphery/ internal cellular distribution (see Figure 6C). Images (representative) are 3D projections of z-stacks collected following plasmolysis with chlorophyll overlay (blue), showing (top to bottom), mCherry-AHA (red), GFP or GFP-SNARE (green) and brightfield. GFP on its own can be constitutively secreted and acidity of apoplast quenches signal resulting in reduced fluorescence observed in confocal images. Scale bar = 20µm. N = 3.

(D) Representative immunoblots, verifying expression of the mCherry-AHA1 (top panel) and GFP-SYP132 (middle panel) in *Nicotiana tabacum* (C, and Figure 6B). Immunoblot analysis used anti-mCherry antibodies to detect mCherry-AHA1 at approx. 133 kDa, and anti-GFP antibodies to detect GFP (control), GFP-SYP132 at approx. 64 kDa and GFP-ΔNH_{abc}-SYP132 at approx. 42 kDa. Total protein in each lane was detected using ponceau stain on immunoblot membrane (bottom panel).

REFERENCES

Haruta M, Sussman MR (2012) The effect of a genetically reduced plasma membrane protonmotive force on vegetative growth of Arabidopsis thaliana Plant Physiology **doi:10.1104/pp.111.189167**

Xia L, Mar Marques-Bueno M, Bruce CG, Karnik R (2019) Unusual Roles of Secretory SNARE SYP132 in Plasma Membrane H(+)-ATPase Traffic and Vegetative Plant Growth. Plant Physiol **180:** 837-858
